# Telomerase RNA-based aptamers restore defective myelopoiesis in congenital neutropenic syndromes

**DOI:** 10.1038/s41467-023-41472-7

**Published:** 2023-09-22

**Authors:** Elena Martínez-Balsalobre, Jesús García-Castillo, Diana García-Moreno, Elena Naranjo-Sánchez, Miriam Fernández-Lajarín, María A. Blasco, Francisca Alcaraz-Pérez, Victoriano Mulero, María L. Cayuela

**Affiliations:** 1 Grupo de Telomerasa, Cáncer y Envejecimiento, Hospital Clínico Universitario Virgen de la Arrixaca, 30120 Murcia, Spain; 2 Instituto Murciano de Investigación Biosanitaria (IMIB) Pascual Parrilla, 30120 Murcia, Spain; 3 Departamento de Biología Celular e Histología, Facultad de Biología, Universidad de Murcia, 30100 Murcia, Spain; 4 Centro de Investigación Biomédica en Red de Enfermedades Raras (CIBERER), ISCIII, 28029 Madrid, Spain; 5 Telomeres and Telomerase Group, Molecular Oncology Program, Spanish National Cancer Centre (CNIO), Melchor Fernández Almagro 3, 28029 Madrid, Spain

**Keywords:** Target identification, Haematological diseases, Long non-coding RNAs, Gene regulation

## Abstract

Telomerase RNA (*TERC*) has a noncanonical function in myelopoiesis binding to a consensus DNA binding sequence and attracting RNA polymerase II (RNA Pol II), thus facilitating myeloid gene expression. The CR4/CR5 domain of *TERC* is known to play this role, since a mutation of this domain found in dyskeratosis congenita (DC) patients decreases its affinity for RNA Pol II, impairing its myelopoietic activity as a result. In this study, we report that two aptamers, short single-stranded oligonucleotides, based on the CR4/CR5 domain were able to increase myelopoiesis without affecting erythropoiesis in zebrafish. Mechanistically, the aptamers functioned as full *terc;* that is, they increased the expression of master myeloid genes, independently of endogenous *terc*, by interacting with RNA Pol II and with the *terc*-binding sequences of the regulatory regions of such genes, enforcing their transcription. Importantly, aptamers harboring the CR4/CR5 mutation that was found in DC patients failed to perform all these functions. The therapeutic potential of the aptamers for treating neutropenia was demonstrated in several preclinical models. The findings of this study have identified two potential therapeutic agents for DC and other neutropenic patients.

## Abstract (web summary)

The RNA component telomerase (*TERC*) has a critical non-canonical role in the formation of the myeloid lineage. Here, the authors show aptamers that mimic the behaviour of the entire *TERC* molecule in myelopoiesis activation.

## Introduction

Telomerase is an RNA-dependent DNA polymerase that synthesizes telomeric repeats at the end of eukaryotic chromosomes to prevent telomeric shortening^1^. This enzyme consists mainly of a protein component (TERT) and an RNA component (*TERC*)^2^. *TERC* has a very complex structure characterized by the presence of three conserved structural domains: the pseudoknot/core domain, which contains the template sequence and is essential for telomerase activity, the conserved regions 4 and 5 (CR4/CR5), which are critical for TERT association, and the ScaRNA domain, which contains the H/ACA and CR7 domains that are responsible for interacting with other telomerase-associated proteins^3,4^.

Telomerase mutations are associated with premature ageing, while telomerase reactivation in adult cells is associated with cancer^5^. However, numerous studies have shown that both components of telomerase complex are involved in several cell signaling pathways without any apparent involvement of its well established function in telomere maintenance^6,7^. Specifically, our laboratory has discovered that *TERC* has an extracurricular role in the regulation of myelopoiesis in both zebrafish and humans^8,9^. In these models, *TERC* behaves as a typical transcription factor that regulates myelopoiesis through regulating the expression of the master myeloid genes *csf3b* (colony-stimulating factor 3b) and *spi1b* (Spi1 proto-oncogene b) in zebrafish and *CSF2* and *SPI1* in humans by recruiting RNA polymerase II (RNA Pol II) to a consensus DNA binding sequence found in their promoters or enhancers^10^. Thus, *terc* overexpression promotes myelopoiesis, increasing the number of neutrophils and macrophages, while *terc* deficiency results in neutropenia and monocytopenia in zebrafish^8,9^. Importantly, the CR4/CR5 domain of *TERC* is essential for performing this novel role in the regulation of myelopoiesis, since a mutation of this domain found in dyskeratosis congenita (DC) patients, but not patient mutations affecting other domains, results in the inability of *TERC* to properly regulate myelopoiesis and to physically interact with RNA Pol II, despite its DNA binding capacity being unaffected^9^. These results might pave the way for the generation of new tools for the treatment of pathologies in which neutropenia is a hallmark.

Neutropenia is a dangerous and potentially fatal disease characterized by a lower than normal number of neutrophils in the circulation, due to reduced production, accelerated elimination or storage problems. Neutropenia can be caused by multiple factors, such as chemotherapy, drugs, infections, autoimmune diseases, bone marrow disorders, chronic idiopathic neutropenia^11^ or genetic disorder (as it happens in DC)^12,13^. DC is an inherited sickness associated with mutations in telomerase components or in telomere-stabilizing components^14^. The principal cause of premature mortality is bone marrow failure, associated with the development of aplastic anemias and myelodysplastic syndromes^15,16^. The incidence of these hematopoietic phenotypes is higher in patients harboring mutations that affect *TERC* than in those patients with other mutations, an observation that cannot be explained by telomere shortening alone^17^. It is tempting to speculate, therefore, that these hematopoietic syndromes are related with the role of *TERC* in myelopoiesis.

The currently available treatment for neutropenia is the administration of granulocyte colony stimulating factor (GCSF, encoded by the *CSF3* gene)^11,18^. GCSF has been approved by the Food and Drug Administration (FDA) for the treatment of congenital and acquired neutropenia and for the mobilization of peripheral hematopoietic progenitor cells for stem cell transplantation^19^. In addition, GCSF therapy has been used in DC patients, being relatively effective in children and young adults suffering this disorder^20–23^. However, while GCSF treatment has some side effects, the main disadvantage is its high cost. Since increased *TERC* expression activates myelopoiesis, precisely through the induction of *GCSF* among other myeloid genes, this could be a strategy for treating neutropenia, monocytopenia or telomeric diseases, such as DC. In addition, RNA synthesis is cheaper compare with GCSF production. Unfortunately, it is not feasible to manipulate *TERC* expression in patients, as it is in zebrafish or cell line models. Therefore, we decided to design a series of RNA aptamers based on the CR4/CR5 domain of *TERC* trying to mimic *TERC* overexpression effect observed in our experimental models.

Aptamers are synthetic single-stranded DNA or RNA sequences that adopt unique three-dimensional structures that allow them to recognize a specific target with high affinity^24^. They have been used as therapeutic agents in several diseases^25,26^. In the present study, several aptamers based on the CR4/CR5 domain of human and zebrafish *TERC* are designed and characterized. The results demonstrate that they behave as endogenous *TERC* enforcing myelopoiesis through the recruitment of RNA Pol II to master myeloid genes. In addition, preclinical zebrafish models of DC and poikiloderma with neutropenia and iPSC human model point to their usefulness to treat congenital neutropenia caused by different genetic alterations.

## Results

### Aptamers were designed based on CR4/CR5 domain of TERC

We have previously shown that *TERC* plays an essential role in myelopoiesis by increasing myeloid gene expression^8,9^. As the CR4/CR5 domain of *TERC* is responsible for this noncanonical function, it was decided to design several aptamers based on the zebrafish CR4/CR5 *terc* domain to test its potential clinical usefulness in blood disorders. The first aptamer was full CR4/CR5 domain, known as *T800* aptamer, and the second one was *T1000* aptamer, consisting of only the P6.1 fork of the CR4/CR5 domain. This fork was selected for study because the mutation G305A in humans (G194A in zebrafish) has been shown to produce aplastic anemia (AA)^27^. As a control, another aptamer was designed from the P6.1 fork but containing the G194A mutation, leading to it being called *T1000*_*mut*_ aptamer (Figs. S1a–S1c). Notably, this mutation drastically altered the aptamer structure (Fig. S1d), suggesting the importance of the tertiary structure of *terc* to perform its noncanonical function in myelopoiesis and anticipating its relevance in DC. As an additional control, we design an aptamer based in the CR7 fork of ScaRNA domain, called *CR7* aptamer, which is not involved in myelopoiesis, since the mutation of this domain found in DC patient was able to increase myelopoiesis as wild type *terc* did^9^ (Figs. S1a–S1d). None of the used aptamers affected embryo development (Table S1).

Mouse and human aptamers were also designed and they had similar structures to the zebrafish ones (Figs. S1c, S1d). In addition, the mutation of the control aptamers (*T1000*_*mut*_) found in AA patients (G305A)^27^ dramatically changed their structure (Figs. S1c, S1d). As an additional control, human *CR7* aptamer was also designed. All the human and mouse aptamers were chemically modified on the sugar ring, replacing the 2′ position with an O-methyl group (OCH3) on the first and last two bases of every aptamer to increase stability and prevent degradation by nucleases^28–30^. Furthermore, they were labeled at their 3′ end by Cy3 fluorescent dye to facilitate tracking.

### Aptamers increase myelopoiesis, without affecting erythropoiesis, in zebrafish

**Fig. 1:**
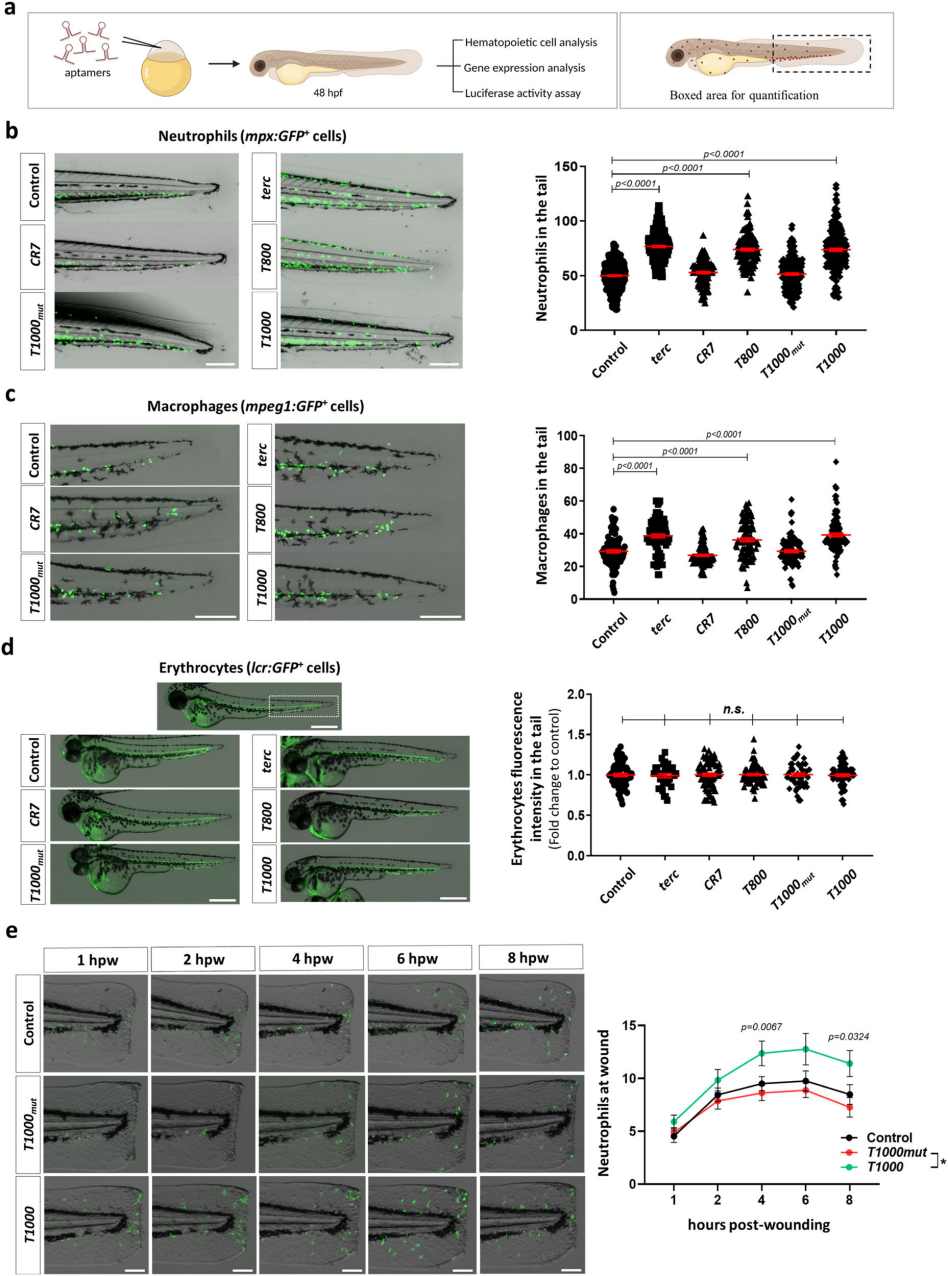
T800 and T1000 aptamers enforce myelopoiesis in zebrafish. **a** General workflow (created with Bio Render). Representative pictures of mpx:GFP (**b**) and mpeg1:GFP (**c**) larvae tail and lcr:GFP (**d**) complete larvae by 48 h post-fertilization (hpf) microinjected with the indicated aptamers. Right panels show quantification of neutrophils (**b**), macrophages (**c**) and erythrocyte fluorescence intensity in the white boxed area (**d**). **e** Representative images of mpx:GFP larvae transected tails from 1 to 8 h post-wounding (hpw) and quantification of neutrophil migration to the wound as neutrophil number mean±s.e.m. for all larvae. **b** Data are average of 5 independent experiments. Each dot represents a single larva (Control, n = 315; *terc*, n = 223; *CR7*, n = 120; *T800*, n = 151; *T1000mut*, n = 224; *T1000*, n = 219). Mean ± SEM for each group is also shown. Statistical analysis according to ordinary 1way ANOVA followed by Dunnett’s multiple comparison test (95% confidence interval). Scale bar: 250 um. **c** Data are average of 3 independent experiments. Each dot represents a single larva (Control, n = 97; *terc*, n = 84; *CR7*, n = 88; *T800*, n = 93; *T1000mut*, n = 92; *T1000*, n = 102). Mean ± SEM for each group is also shown. Statistical analysis according to ordinary 1way ANOVA followed by Dunnett’s multiple comparison test (95% confidence interval). Scale bar: 250 um. **d** Data are average of 3 independent experiments. Each dot represents a single larva (Control, n = 72; *terc*, n = 34; *CR7*, n = 75; *T800*, n = 71; *T1000mut*, n = 46; *T1000*, n = 75). Mean ± SEM for each group is also shown. *n.s*., not significant, p > 0.05 according to ordinary 1way ANOVA followed by Dunnett’s multiple comparison test (95% confidence interval). Scale bar: 500 um. **e** Data are shown as mean ± SEM of 3 independent experiments (Control, n = 30; *T1000mut*, n = 38; *T1000*, n = 43). Differences are statistically significant between *T1000mut* and *T1000* groups. Statistical analysis according to mixed-effects analysis followed by Tukey’s multiple comparison test (95% confidence interval). Scale bar: 100 um. Image was created with BioRender.com by co-Authors. Source data are provided as a Source Data file.

To evaluate the effect of *terc*-derived aptamers on hematopoiesis, they were microinjected into zebrafish embryos at the one-cell stage, and the larvae were analyzed 48 h later (Fig. 1a). The levels of every aptamer were checked by RT-qPCR in every single experiment and if they were not detected, the experiment was discarded (Fig. S2a).

*T800* and *T1000* aptamers were able to increase neutrophil and macrophage numbers at full *terc* level, assayed in *mpx:GFP* and *mpeg1:GFP* transgenic zebrafish lines, respectively (Fig. 1b, c). However, the *CR7* aptamer failed to increase neutrophil and macrophage numbers, confirming the specificity of the observed effects with *T800* and *T1000* aptamers and supporting our hypothesis that individual domains of *terc* can perform its function in myelopoiesis in the same way as the full molecule. Importantly, the *T1000*_*mut*_ aptamer failed to affect the number of myeloid cells, demonstrating that this point mutation has a drastic effect on *T1000* function, suggesting the importance of a correct tertiary structure for its function in the regulation of myelopoiesis and confirming the relevance of this mutation in DC pathology. Furthermore, there were no differences in the erythrocyte number (*lcr:GFP* positive cells) of larvae microinjected with different aptamers or full *terc* (Fig. 1d), confirming previous results obtained with full *terc*^8^. Together, these results indicate that aptamers promote myelopoiesis without affecting erythropoiesis, an observation that is crucial for their possible therapeutic use.

As *terc* regulates neutrophil numbers in zebrafish larvae through fine-tuning the expression levels of the main myeloid (*spi1b*) and erythroid (*gata1a*) transcription factors and *csf3b* mRNA expression^8^, the transcript levels of these genes were analyzed by RT-qPCR in larvae treated with the different aptamers. The results showed that although the *T800* and *T1000* aptamers were able to increase *spi1b* and *csf3b* transcript levels and the *spi1b*/gata1 ratio in the same way as full *terc*, the *CR7* and *T1000*_*mut*_ negative controls failed to do so (Fig. S2b), which would explain the observed increase in neutrophil and macrophage numbers (Fig. 1b).

As aptamers may be recognized as foreign RNA by innate immune system, we checked the expression of several inflammatory and interferon gene markers, including interferon-stimulated gene 15 (*isg15*), NLR family pyrin domain containing 3 (*nlrp3*) and tumor necrosis factor α (*tnfa*). The results showed that the transcript levels of none of these genes were affected by the aptamers (Fig. S2c). In addition, neutrophil activity, assayed by a recruitment assay to wound, was also unaffected (Fig. 1e). Thus, although the neutrophil number at the injury site was higher at all time points analyzed in *T1000* aptamer treated larvae compared with *T1000*_*mut*_ aptamer or without treatment, the kinetics of the recruitment was similar in both groups (Fig. 1e).

### Aptamers physically bind to DNA and to RNA pol II

**Fig. 2:**
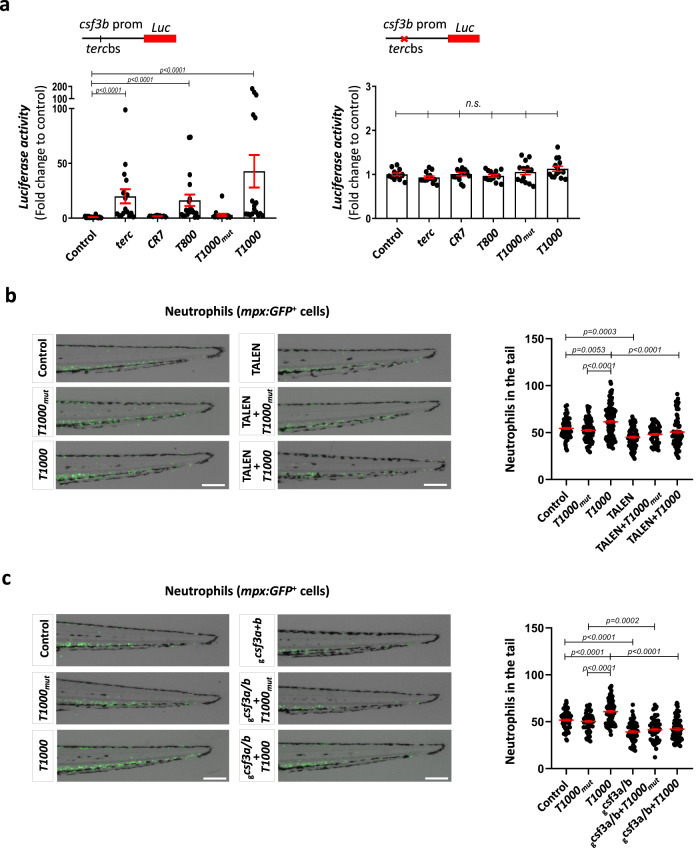
T1000 aptamer enforce myelopoiesis by activating *gcsf3b tercbs* and *gcsfa/b* expression. **a** Luciferase assay using wild type or mutant *tercbs* of *csf3b* promoter. Luciferase activity was normalized to Renilla activity and the results shown as fold change relative to control. **b**, **c** Representative images and neutrophil quantification of 72 hpf Tg(mpx:GFP) tail larvae. Larvae were microinjected with the indicated aptamer in combination with TALEN targeting the *gcsf3b tercbs* promoter (**b**) or gRNAs against *csf3a/b* genes (**c**). Data are average of 3 independent experiments. **a** Each dot represents a biologically independent sample consisting of a pool of 20 larvae (Left: Control, n = 19; *terc*, n = 16; *CR7*, n = 10; *T800*, n = 19; *T1000mut*, n = 15; *T1000*, n = 18. Right: Control, n = 13; *terc*, n = 14; *CR7*, n = 14; *T800*, n = 14; *T1000mut*, n = 14; *T1000*, n = 14). Bars represent mean ± SEM for each group. *n.s*., not significant, p > 0.05 according to Kruskal-Wallis followed by Dunn’s multiple comparison test (95% confidence interval). **b** Each dot represents a single larva (Control, n = 76; *T1000mut*, n = 84; *T1000*, n = 109; TALEN, n = 75; TALEN+*T1000mut*, n = 51; TALEN+*T1000*, n = 77). Mean ± SEM for each group is also shown. Statistical analysis according to ordinary 1way ANOVA followed by Sidak’s multiple comparison test (95% confidence interval). Scale bar: 200 um. **c** Each dot represents a single larva (Control, n = 59; *T1000mut*, n = 66; *T1000*, n = 81; _g_*csf3a*+*b*, n = 56; _g_*csf3a*+*b* +*T1000mut*, n = 59; _g_*csf3a*+*b* +*T1000*, n = 71). Mean ± SEM for each group is also shown. Statistical analysis according to ordinary 1way ANOVA followed by Sidak’s multiple comparison test (95% confidence interval). Scale bar: 200 um. Source data are provided as a Source Data file.

It has been shown that *terc* regulates the expression of myeloid genes by binding to consensus sequences^10^, called *terc* binding sites (*terc*bs), and by recruiting the transcriptional machinery^9^. As these sequences are found in the *csf3b* promoter, it was cloned into a luciferase reporter for in vivo transcription activity assays. Fig. 2a shows that, while the *T800* and *T1000* aptamers were both able to increase wild type *csf3b* promoter activity at similar levels to full *terc*, the promoter lacking the *terc*bs did not change upon treatment by *terc* or aptamers. As expected, the *CR7* and *T1000*_*mut*_ aptamers were not able to increase the activity of either wild type or mutant *csf3b* promoters. Furthermore, either genetic deletion of *tercbs* of *csf3b* promoter (Fig. 2b) or Csf3a/Csf3b deficiency (Fig. 2c) impaired the induction of myelopoiesis by *T1000* aptamer. This demonstrated that the *terc*bs region was necessary for *csf3b* expression regulation by aptamers, as occurs with *terc*, confirming that aptamers regulate myelopoiesis using the same mechanism as full *terc*.

**Fig. 3:**
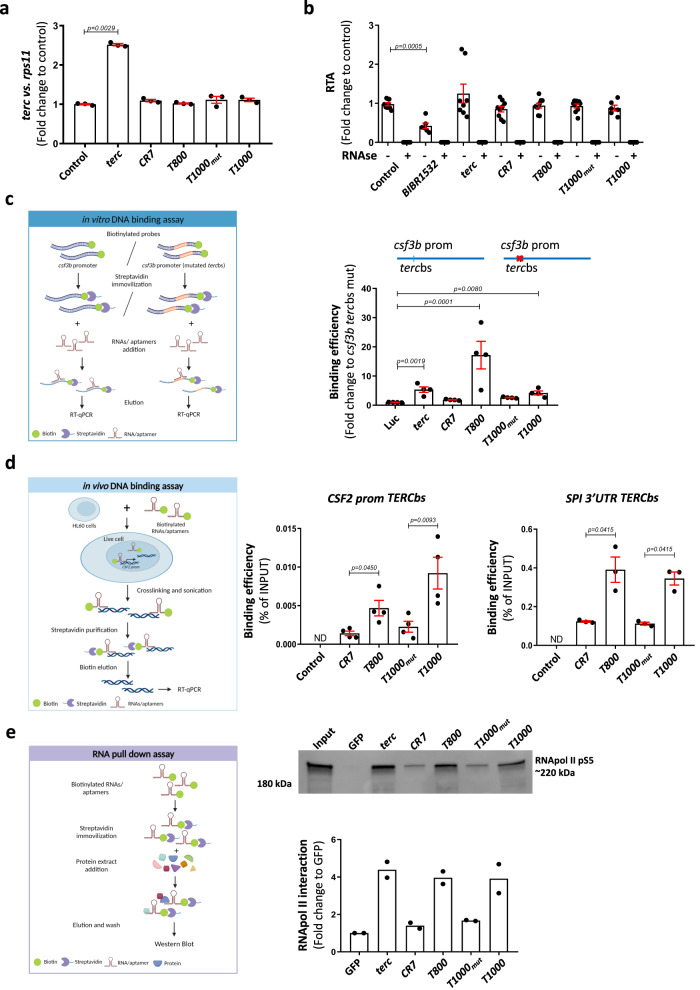
Aptamers physically bind to DNA and to RNApolII. **a** *terc* RNA levels determined by RT-qPCR in 48hpf larvae microinjected with full *terc* or the indicated aptamers. Gene expression was normalized to *rps11* and then to the control sample. **b** Relative telomerase activity (RTA) measured by qPCR in protein extracts of 48 hpf larvae in the indicated conditions. **c** RT-qPCR of in vitro DNA-binding assay eluates showing the affinity for the wildtype *csf3b* promoter related to the *csf3b* promoter with mutated *terc* binding site (*terc*bs), and compared to Luc control. **d** RT-qPCR of in vivo DNA-binding assay eluates showing the affinity for the *CSF2* promoter *TERC*bs or the *SPI* 3’UTR *TERC*bs. **e** Western blot and quantification of aptamer RNA pulldown eluates using anti-phospho-serine 5 RNA Pol II antibody. **a** Data are average of 3 biologically independent samples. Each dot represents a pool of 20 larvae. Bars represent mean ± SEM. Statistical analysis according to Kruskal-Wallis followed by uncorrected Dunn’s test (95% confidence interval). **b** Data are average of 3 independent experiments. Each dot represents a biologically independent sample consisting of a pool of 25 larvae (Control, n = 6; BIBR1532, n = 6; *terc*, n = 8; *CR7*, n = 6; *T800*, n = 9; *T1000mut*, n = 9; *T1000*, n = 9). Bars represent mean ± SEM. Statistical analysis according to Kruskal-Wallis followed by uncorrected Dunn’s test (95% confidence interval). **c** Data are average of 4 independent experiments. Bars represent mean ± SEM. Statistical analysis according to Kruskal-Wallis followed by Dunn’s multiple comparison test (95% confidence interval). **d** Data are average of 3-4 independent experiments. Bars represent mean ± SEM. Statistical analysis according to Kruskal-Wallis followed by uncorrected Dunn’s test (95% confidence interval). **e** Data are average of 2 independent experiments. Each dot represents a biologically independent sample consisting of a pool of 25 larvae. Bars represent mean. Images were created with BioRender.com by co-authors. Source data are provided as a Source Data file.

As the simplest theory to explain the action mechanism of aptamers is an indirect effect through the modulation of endogenous *terc*, the RNA levels of *terc* were measured by RT-qPCR, and the results showed that its transcript levels were unaltered by aptamers (Fig. 3a). In was also thought interesting to know if aptamers affect telomerase activity, and, while it was found that neither aptamers nor full *terc* affected telomerase activity, the telomerase activity inhibitor BIBR1532 reduced it more than 50%, as was expected (Fig. 3b). Therefore, the ability of aptamers to promote myelopoiesis is not mediated through the regulation of endogenous *terc* or telomerase activity.

The ability of aptamers to increase neutrophil and macrophage numbers through engaging the *terc*bs in the *csf3b* promoter led us to hypothesize that aptamers were acting as *terc*. To confirm the direct binding of aptamers to DNA, an aptamer-DNA binding assay was performed to evaluate whether aptamers were able to bind to putative *terc*bs in vitro. For this, a dsDNA biotinylated probe of *csf3b* upstream regulatory sequences (100 bp) containing *terc*bs was incubated with aptamers and *terc*, as positive control, or luciferase mRNA (*luc*), as negative control. In parallel, the same experiment was performed with a *csf3b* promoter probe with mutant *terc*bs. The results showed that *terc* was able to bind to the *csf3b* probe with very high affinity compared to the control probe (*luc*) (Fig. 3c), confirming the relevance of *terc*bs for *terc* binding to DNA^9^. Similarly, the *T800* aptamer robustly bound to wild type *csf3b* probe compared with the rest of aptamers and full *terc*, while *T1000* and *T1000*_*mut*_ interacted weakly with the probe, all interactions being *terc*bs-dependent (Fig. 3c). However, both human *T800* and *T1000* aptamers interacted in vivo with similar affinities with the *TERC*bs of *CSF2* and *SPI1* promoters in HL-60 cells (Fig. 3d). These results show that *T800* and *T1000* aptamers can directly bind the *terc*bs.

These results prompted us to check whether the aptamers were also able to interact with RNA Pol II, as could *terc*^9^. For this, an RNA pulldown procedure was performed using biotinylated aptamers incubated with protein extract from 5-day-old zebrafish larvae. Biotinylated *terc*, as positive control, and biotinylated GFP mRNA, as negative control, were also included. It was found that both *T800* and *T1000* aptamers interacted with RNA Pol II with similar efficiency to that of *terc* (Fig. 3e). As expected, the negative control aptamers *CR7* and *T1000*_*mut*_ did not show a statistically significant interaction with RNA Pol II. Therefore, the aptamer action mechanism seems to be similar to that of *terc*, since aptamers are able to bind to DNA and to RNA pol II.

### Aptamers rescue neutropenia in zebrafish disease models of dyskeratosis congenita and poikiloderma with neutropenia

**Fig. 4:**
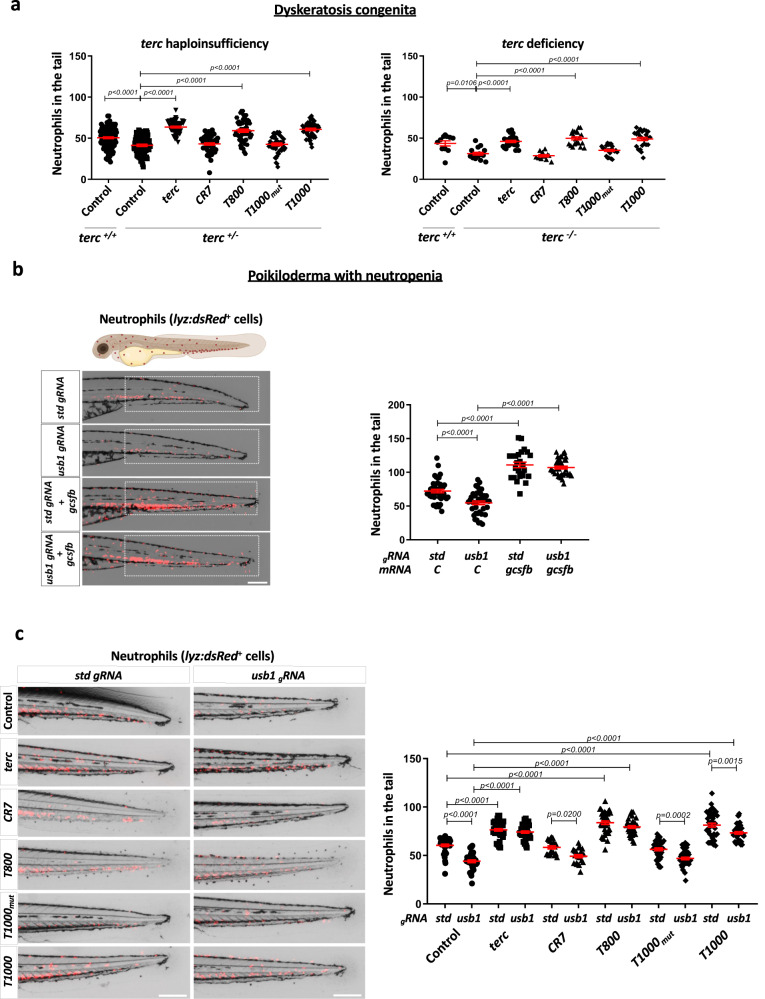
Aptamers rescue neutropenia in zebrafish models of DC and PN. **a** Neutrophils quantification at 48 hpf in *terc* +/- (obtained from mpx:GFP fish outcrossed with *terc* -/-) and *terc* -/- (obtained from a *terc* -/- incross) larvae microinyected with the indicated RNAs. *terc* -/- larvae were stained with TSA reagent to visualize neutophils. **b** Representative pictures and neutrophils (dsRed positive cells) quantification at 72 hpf in the tail of lyz:dsRED larvae microinjected with *usb1* or std gRNA plus recombinant Cas9 in presence or absence of *gcsfb* mRNA. The white boxes show the quantified area. **c** Representative images and neutrophils quantification at 72 hpf in lyz:dsRED larval tail microinjected with *usb1* or std gRNA plus recombinant Cas9 and the indicated aptamers. **a** Each dot represents a single larva (Left: +/+ Control, n = 126; +/- Control, n = 96; +/- *terc*, n = 48; +/- *CR7*, n = 52; +/- *T800*, n = 57; +/- *T1000mut*, n = 52; +/- *T1000*, n = 54. Right: +/+ Control, n = 11; -/- Control, n = 16; -/- *terc*, n = 26; -/- *CR7*, n = 11; -/- *T800*, n = 20; -/- *T1000mut*, n = 24; -/- *T1000*, n = 25). Mean ± SEM for each group is also shown. Left: Statistical analysis according to ordinary 1way ANOVA followed by Dunnett’s multiple comparison test (95% confidence interval). Right: Statistical analysis according to Kruskal-Wallis followed by Dunn’s multiple comparison test (95% confidence interval). **b** Data are average of 3 independent experiments. Each dot represents a single larva (Control+_g_std, n = 41; Control+_g_*usb1*, n = 40; *gcsfb*+_g_std, n = 25; *gcsfb*+_g_*usb1*, n = 37). Mean ± SEM for each group is also shown. Statistical analysis according to ordinary 1way ANOVA followed by Dunnett’s multiple comparison test (95% confidence interval). Scale bar: 250 um. **c** Data are average of 3 independent experiments. Each dot represents a single larva (Control+_g_std, n = 42; Control+_g_usb1, n = 36; *terc*+_g_std, n = 46; *terc*+_g_usb1, n = 39; *CR7*+_g_std, n = 21; *CR7*+_g_*usb1*, n = 22; *T800*+_g_std, n = 39; *T800*+_g_*usb1*, n = 32; *T1000mut*+_g_std, n = 38; *T1000mut*+_g_*usb1*, n = 38; *T1000*+_g_std, n = 42; *T1000*+_g_*usb1*, n = 39). Mean ± SEM for each group is also shown. Statistical analysis according to ordinary 1way ANOVA followed by Sidak’s multiple comparison test (95% confidence interval). Scale bar: 250 um. Image was created with BioRender.com by co-Authors. Source data are provided as a Source Data file.

*TERC* haploinsufficiency generates telomeropathies, such as DC, because telomerase is unable to maintain telomere length in tissues that need constant renewal, such as the hematopoietic system^16^. Therefore, it was decided to test aptamers in heterozygous *terc* (*terc* ^*+/-*^) and homozygous (*terc* ^-*/-*^) zebrafish lines, both of which show telomere shortening and neutropenia at 48 hpf (Fig. 4a). Notably, the *T800* and *T1000* aptamers were able to rescue neutropenia of both lines, in the same way as *terc* (Fig. 4a). This result is of highly relevance from a therapeutic point of view, since it suggests that aptamers could be of use to rescue neutropenia in DC patients with *TERC* haploinsufficiency and short telomeres.

In order to further support the usefulness of the clinical application of the *terc*-based aptamers, it was decided to test them in another zebrafish disease model of neutropenia. One of these models is a very rare autosomal recessive genodermatosis characterized by hypopigmentation or hyperpigmentation of different skin areas, skeletal defects and bone marrow alterations, called poikiloderma with neutropenia (PN)^31,32^. PN is caused by mutations in the U6 biogenesis 1 (*USB1*) gene, a 3′‐5′ exonuclease able to post‐transcriptionally remove uridine and adenosine nucleosides from the 3′ end of the spliceosomal U6 small nuclear RNA. Nearly half of PN patients develop myelodysplastic syndrome and acute myeloid leukemia in the second decade of life, followed by bone marrow failure syndrome predisposing patients to cancer^33^. The symptoms may be similar to those of DC, although the genetic causes are completely different^34^. The treatment of this disease is based on alleviating the symptoms, so, in cases of neutropenia, GCSF is administered^35^. To generate the zebrafish model of PN, genetic inactivation of *usb1* with CRISPR-Cas9 was performed in the *lyz:dsRED* instead of the *mpx:GFP* zebrafish line, since it has been reported that *usb1* deficiency results in alteration of several genes involved in neutrophil differentiation and development, including *mpx*^32^. It was confirmed that the genetic inhibition of *usb1* results in a decreased number of neutrophils in zebrafish (Fig. 4b). Moreover, the administration of *gcsf* mRNA rescued neutropenia in this model (Fig. 4b), as occurs in PN patients. When the aptamers were used, interestingly, both *T800* and *T1000* were able to rescue neutropenia of the PN model, at similar levels to *terc*, while the negative controls *CR7* and *T1000*_*mut*_ failed to do so (Fig. 4c). This result suggests that *T800* and *T1000* aptamers may be an effective treatment of human neutropenia that responds to GCSF treatment.

### Aptamers rescue defective myeloid differentiation of mouse bone marrow cells and iPSCs from DC patients

*T800* and *T1000* zebrafish aptamers have been shown to increase the number of neutrophils in both healthy and neutropenic fish models, probably through the recruitment of RNA Pol II to myeloid gene promoters, such as spi*1* and *csf3b*, thus enhancing their expression. These results, together with the absence of apparent side effects in the tested models, suggest their usefulness for treating human diseases. Consequently, the same aptamers, but based on the mouse or human telomerase RNA structure, were synthetized (Figs. S1c and S1d) and tested in mouse and human hematopoietic colony-forming cell assays, since the noncanonical function of *TERC* in myelopoiesis is highly conserved between mammals and zebrafish.

**Fig. 5:**
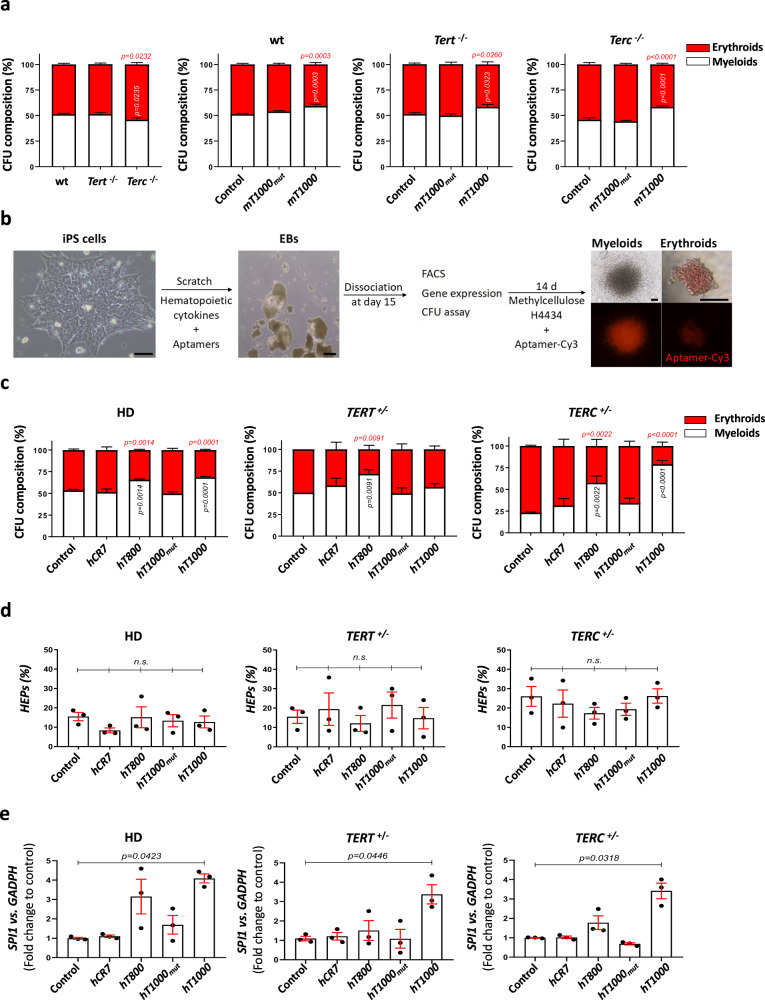
Aptamers rescue defective myelopoiesis in mouse and iPSC from DC patients. **a** Left graph; comparison of the composition of erythroid and myeloid colonies assayed by colony assays, using whole bone marrow cells from mice of the indicated phenotype. Middle and right graphs; composition of erythroids and myeloids assayed by colony assays, using whole bone marrow cells from mice of the indicated phenotype an incubated with the mouse *T1000* aptamer. N = 7 for wt and *Terc* -/- mice and N = 5 for *Tert* -/- mice. **b** Induced-pluripotent stem cell (iPSC) workflow. **c** Composition of erythroids and myeloids assayed by colony assays, using embryonic bodies (EBs) derived from the indicated iPSC lines treated with different aptamers. **d** Quantification of the hemogenic progenitors (HEPs) population (CD31 + /CD34 + /CD45-) by flow cytometry at day 15 in EBs. **e** mRNA levels of *SPI1* assayed by RT-qPCR in the different conditions. The expression is normalized to *GAPDH* and relative to control sample. **a** Data are average of at least 5 biologically independent samples (wt, n = 7; *Tert*-/-, n = 5; *Terc*-/-, n = 7). Bars represent mean ± SEM. Statistical analysis according to 2way ANOVA followed by Dunnett’s multiple comparison test (95% confidence interval). **c** Data are average of 3 biologically independent samples. Bars represent mean ± SEM. Statistical analysis according to 2way ANOVA followed by Dunnett’s multiple comparison test (95% confidence interval). **d** Data are average of 3 biologically independent samples represented by dots. Bars represent mean ± SEM for each group. *n.s*., not significant, p > 0.05 according to Kruskal-Wallis followed by Dunn’s multiple comparison test (95% confidence interval). **e** Data are average of 3 biologically independent samples represented by dots. Bars represent mean ± SEM. Statistical analysis according to Kruskal-Wallis followed by Dunn’s multiple comparison test (95% confidence interval). Source data are provided as a Source Data file.

Unexpectedly, bone marrow cells from *Terc*^-/-^ showed weak, but statistically significant, myeloid differentiation defect compared with wild type and *Tert*^-/-^ cells (Fig. 5a and S3a). Addition of aptamer *mT1000* increased the number of CFU-GM at the expenses of BFU-E in the 3 genotypes (Fig. 5a, S3a). We next evaluated the ability of aptamers to regulate myelopoiesis in humans using two iPSC lines derived from DC patients and one derived from a healthy donor (HD). iPSCs from DC patients harbored either a heterozygous mutation in the CR4/CR5 domain of *TERC* (nG319A)^36^ or a heterozygous pathogenic missense point mutation in TERT (A716V)^9^. iPSCs were differentiated into hematopoietic cells through embryonic body (EB) formation, which are three-dimensional cell aggregates that can differentiate into cells of all three germ layers. Seven days after differentiation started, aptamers were added to the medium before carrying out colony-forming unit (CFU) assays and flow cytometry and gene expression analysis (Fig. 5b)^37^. We firstly checked by RT-qPCR that aptamers were taken up by cells (Figs. S4a–S4c). As we recently reported^9^, iPSC harboring the *TERC* mutation showed impaired myelopoiesis (Fig. 5c, S3b). Strikingly, *hT800* and *hT1000* aptamers were both able to fully rescue the defective myeloid differentiation of *TERC* mutant iPSC by increasing the percentage of myeloid colonies (CFU-G/M/GM) to 50% (Fig. 5c). However, aptamer administration did not affect the percentage of hemogenic progenitors (HEPs) (CD31^+^/CD34^+^/CD45^-^) (Fig. 5d), hematopoietic progenitors (HPC) (CD31^-^/CD34^+^/CD45^+^) or mature blood cells (CD34^-^/CD45^+^) (Figs. S3c, S3d, S3e). As the CFU potential robustly correlated with HEPs numbers^37^ and aptamers did not change the number of progenitors, aptamers seem to act by favoring their differentiation into CFU-GM at the expenses of erythroid colonies (BFU-E). This is consistent with the ability of both *hT800* and *hT1000* aptamers to significantly rescue the reduced SPI*1* transcript levels observed in the EB obtained from *TERC*-deficient iPSCs (Fig. 5e and S5a). However, although none of the aptamers significantly affected the transcript levels of *GATA1*, *CSF3*, *TERT* and *TERC* in the EBs, the administration of *hT800* and *hT1000* aptamers showed a tendency to increase the reduced mRNA levels found in the EB derived from *TERC*-deficient iPSCs (Figs. S5a–S5e). Together, these data further support the clinical application of the *T800* domain-based aptamers to treat neutropenia of DC patients with mutations affecting the CR4/CR5 domain of *TERC*.

## Discussion

Aptamers are of high interest for biomedicine application, such as treating cancer, immune diseases, acute myeloid leukemia, metabolic skeletal disorders or thrombosis, due to their ability to bind to specific biological molecules^24^. Aptamers are selected through an in vitro process named Systematic Evolution of Ligands by Exponential enrichment (SELEX)^38^. However, in our study, we took advantages of our extensive knowledge of *TERC* to design several aptamers. Hence, we have recently discovered that *TERC* behaves as a typical transcription factor, despite being a lncRNA^9^. On the one hand, it is able to recognize a specific binding sequence on the DNA and, on the other hand, to physically interact with RNA Pol II, the main component of the transcriptional machinery, in both zebrafish and humans. Reduced levels of *TERC* in human myeloid progenitors resulted in a decreased occupancy at transcription start sites of key myeloid genes by RNA Pol II, and thus, lower levels of myeloid transcripts^9^. Additionally, we have demonstrated that the CR4/CR5 domain is indispensable for this novel, noncanonical function of *TERC*, since a *TERC* molecule harboring a mutation in the CR4/CR5 domain found in DC patients is unable to regulate the expression of myeloid genes, whereas mutations of DC patients in other domains do not affect this function.

This crucial importance of the CR4/CR5 domain in myelopoiesis led us to try to develop new therapeutic strategies for DC patients, particularly those harboring mutations that affect the CR4/CR5 domain. For this purpose, an array of aptamers was designed based on the sequence of the CR4/CR5 domain. This tool was chosen because of its multiple advantages in terms of specificity, immunogenicity, cost and simplicity of synthesis^24^. The *T800* (CR4/CR5 full domain) and *T1000* (P6.1 fork) were selected as therapeutic aptamers while the same sequence, but with the mutation present in DC patients (G305A for *T1000* aptamer) was selected as control. As an additional control and to further demonstrate the relevance and autonomy of the CR4/CR5 domain to regulate myelopoiesis, we used the aptamer *CR7* which consists on the ScaRNA domain sequence. Surprisingly, we observed that wild type CR4/CR5-based aptamers were able to increase the number of neutrophils and the expression of the master myeloid genes *  spi1b* and *csf3b* in the zebrafish, just as *terc* did, while all control aptamers failed to do so. Importantly, the aptamers did not show toxic effects in zebrafish, and failed to induce inflammation and to alter neutrophil recruitment to wound. Mechanistically, aptamers function in the same way as full *terc*: (i) being able to increase the number of monocytes and neutrophils, without affecting the number of erythrocytes; (ii) increasing the promoter activity of *csf3b* in vivo by binding to *terc*bs; (iii) directly interacting with DNA in vitro and in vivo, such affinity being enhanced when *terc*bs were present; (iv) physically binding to RNA Pol II; and (v) they exerting all these effects independently of endogenous *terc*, since they did not affect the expression of *terc* and can perform their function in myelopoiesis in *terc*-deficient zebrafish models. These results not only show the therapeutical potential of these molecules to treat neutropenia but also demonstrate that the CR4/CR5 domain of *terc* regulates hematopoiesis independently of the rest of its domains.

To demonstrate the therapeutic use of the developed aptamers, bearing in mind that the noncanonical function of *TERC* in myelopoiesis is evolutionarily conserved^9^, homologous molecules were designed based on the mouse and human *TERC* sequences. It was found that both *hT800* and *hT1000* aptamers were able to slightly increase myelopoiesis in HD and TERT-deficient iPSCs but, more importantly, that they fully restored defective myelopoiesis in *TERC*-deficient iPSCs, probably by restoring normal transcript levels of *SPI1*, which were found to be lower, together with those of *CSF3*, in *TERC*-deficient EBs. These low levels of *SPI1* and *CSF3* in *TERC*-deficient EBs, but not in their TERT-deficient counterparts, is surprising because the culture medium contained GCSF, which apparently failed to restore defective granulopoiesis of *TERC*-deficient cells. Importantly, aptamer administration did not alter *GATA1* mRNA levels or the number of HPCs in EBs, so it seems that *TERC* acts by fine-tuning SPI1 and GATA1 levels in HPCs in order to properly balance the production of blood cells, as do other master regulators, such as the inflammasome^39^ and TIF1γ^40^, among others. Importantly, these results, together with the observed lack of effect of aptamer administration in zebrafish erythropoiesis, suggest that aptamers might restore defective myelopoiesis in neutropenic patients without affecting erythropoiesis, an obvious concern in their clinical application. Although further studies in primary CD34^+^ cells from DC patients with mutation of the CR4/CR5 will surely further demonstrate its potential use to treat neutropenia, our study with zebrafish models of PN disease^31,32^, strongly suggests its wide therapeutic potential in treating neutropenic congenital diseases caused by different mutations, beside those caused by mutations in *TERC* or telomeric proteins. Unexpectedly, although *Terc*-deficient bone marrow cells hardly recapitulated the myelopoiesis defects observed in zebrafish larvae and human iPSCs, the *Terc*-based aptamers were able to rescue these defects in all species.

An important observation of the present study is that although the *T1000* aptamer showed reduced DNA-binding activity in vitro, both *T800* and *T1000* aptamers showed similar DNA-binding activity in vivo and ability to promote myelopoiesis in zebrafish and human. This suggests that the CR4/CR5 domain contains important nucleotides outside the P6.1 fork involved in the interaction with *terc*bs. While beyond the scope of the present study, we consider it would be interesting to map these nucleotides to further understand how *TERC* binds to its canonical target in the DNA^10^. It is also likely that other factors, which were only present in the in vivo assay, are important for the interaction between *TERC* and DNA. Whatever the outcome, the small size of *T1000* makes it ideal as a potential therapeutic tool for clinical trial, since its tissue penetration capacity is greater, and the cost of its synthesis is much lower. In such clinical trials, it would also be interesting to compare the aptamers with the routine administration of GCSF, in terms of efficacy and cost, since Filgrastim (the generic name of commercial GCSF) and several biosimilar products, such as Lenograstim and Pegfilgrastim, have high cost (from €800 to €1800 range per patient and treatment cycle)^41^. Furthermore, we anticipate the usefulness of the developed aptamers for the treatment of chemotherapy-induced neutropenia in cancer patients, where post-chemotherapy GCSF treatment accounts for a large percentage (from 26 to 39%) of the total medical costs^42^.

In summary, based on our knowledge of the noncanonical function of *TERC* in the regulation of myelopoiesis, and in particular of its CR4/CR5 domain, the two aptamers that we have designed and characterized are able to perform the same function in myelopoiesis as the complete *TERC* molecule and to restore defective myelopoiesis in zebrafish, mouse and human models of congenital neutropenia caused by different genetic alterations, such as DC and PN.

## Methods

### Ethics statement

The performed experiments comply with the Guidelines of the European Union Council (Directive 2010/63/EU) and the Spanish RD 53/2013. Experiments and procedures were performed as approved by the Bioethical Committees of the University of Murcia (approval numbers #75/2014, #216/2014 and 395/2017).

### Animals

Zebrafish (*Danio rerio* H.) specimens were obtained from the Zebrafish International Resource Center and mated, staged, raised and processed using standard procedures^43^. The transgenic lines *Tg(mpx::eGFP)*^i113^ (*mpx*:*GFP* for simplicity)^44^, *Tg(mpeg1:eGFP)*^*gl22*^ (*mpeg1:GFP* for simplicity)^45^, *Tg(lcr:eGFP)*^*cz3325*^ (*lcr:GFP* for simplicity)^46^ and *Tg(lyz:dsRED)*^*nz50*^ (*lyz:dsRED* for simplicity)^47^, were previously characterized.

### Aptamers and RNA injection

Aptamers were synthesized as a single-stranded RNA oligos unmodified or with modifications to increase stability or to add biotin at 3’end by Sigma-Aldrich, or by Horizon Discovery in the case of *T800* aptamer. The *terc* and *csf3b* RNAs were in vitro transcribed with the mMESSAGE mMACHINE kit (Ambion) and purified with phenol chloroform. Both mRNA and aptamers were mixed in a microinjection buffer (0.5 × Tango buffer and 0.05% phenol red solution) and microinjected into the yolk sac of one-cell stage embryos using a Narishige IM300 microinjector (0.5-1 nl per embryo). The amount of *terc* and *csf3b* injected was 200 and 100 pg/egg, respectively. The amount of aptamers injected was 605 pg/egg for *T800*, 337 pg/egg for *CR7* and 138 pg/egg for *T1000* and *T1000*_*mut*_, in order to achieve the same number of molecules per egg in all the aptamers used.

### Zebrafish blood cell count

Eggs of the one-cell stage from the transgenic lines *mpx:GFP, mpeg1:GFP*, *lcr:GFP* and *lyz:dsRED* were microinjected with the different aptamers or *terc*. Neutrophils (*mpx:GFP* or *lyz:dsRed* positive cells), and macrophages (*mpeg1:eGFP* positive cells) of the tail region of 48 or 72 hpf larvae were counted under a Leica M205 FA fluorescence stereo microscope. To quantify the erythrocytes (*lcr:GFP* positive cells), the larvae were photographed with Leica M205 FA fluorescence microscope equipped with a DFC365FX camera (Leica) and the total fluorescence intensity of the tail region was quantified with Leica Application Suite X (LAS X) software.

### Zebrafish neutrophil staining

Zebrafish embryos (48 hpf) were fixed for 2 h at room temperature (RT) in 4% paraformaldehyde (PFA). Fixed larvae were briefly washed twice in PBS and incubated in 1:50 TSA Cyanine5 (TSA TM-Plus Fluorescein kit, Perkin Elmer, UK) without light for 10 min at 28 °C. Neutrophils were specifically fluorescein stained and then counted under a Leica M205 FA fluorescence stereo microscope equipped with a DFC365FX camera (Leica).

### Aptamer quantification

Total RNA was extracted from larvae tails with TRIzol reagent (Thermo Fisher Scientific) using the Direct-zol RNA Miniprep kit (Zymo Research) and treated with DNAse following the manufacturer’s instructions. cDNA was generated with the miScript II RT kit (Qiagen), following the manufacturer’s instructions. Real-time qPCR was performed with a StepOnePlus instrument (Applied Biosystems), using miScriptSYBR Green PCR kit (Perfect Real Time) (Qiagen), containing a reverse common primer. Reaction mixtures were incubated for 10 min at 95 °C, followed by 40 cycles of 15 s at 95 °C, 1 min at 60 °C, and finally 15 s at 95 °C, 1 min at 60 °C and 15 s at 95 °C. For each sample, gene expression was normalized to *U6* snRNA, using the comparative Ct method (2^-ΔΔCt^). The forward primers used are shown in Table S1. In all cases, each PCR was performed with triplicate samples and repeated in every aptamer microinjection experiment.

### Gene expression analysis

Total RNA was extracted from larvae tails with TRIzol reagent (Thermo Fisher Scientific) using Direct-zol RNA Miniprep kit (Zymo Research) following the manufacturer’s instructions. RNA was treated with DNase I, RNAsa free (Qiagen) on the column. A SuperScript™ VILO™ cDNA Synthesis Kit (Invitrogen) was used to synthesize first-strand cDNA following the manufacturer’s instructions. Real-time PCR was performed with a StepOnePlus instrument (Applied Biosystems) using SYBR® Premix Ex Taq™ (Perfect Real Time) (Takara). Reaction mixtures were incubated for 30 s (sec) at 95 °C, followed by 40 cycles of 5 sec at 95 °C, 20 sec at 60 °C, and finally a melting curve protocol. Ribosomal protein S11 (*rps11*) or glyceraldehyde 3-phosphate dehydrogenase (*GAPDH*) content in each sample was used for normalization of zebrafish or human mRNA expression, respectively, using the comparative Ct method (2-∆Ct). The primers used are shown in Table S1. In all cases, each PCR was performed with triplicate samples and repeated with at least two independent samples.

### Recruitment assay

At 3 dpf, Tg(*mpx::GFP*) injected with *T1000* or *T1000mut* aptamers and uninjected as control larvae were anesthetized in tricaine and complete transection of the tail was performed with a disposable sterile scalpel. Then, they were mounted in 1% (wt/vol) low-melting-point agarose dissolved in egg water. The images were captured at the selected times while animals were kept in the agarose matrixes with the added medium at 28.5 °C. All images were acquired with the integrated camera on the stereomicroscope and were used for subsequently counting the number of neutrophils recruited to the wound area, established between the arterio-venous loop and the end of the tail.

### Telomerase activity assay

A real-time quantitative telomeric repeat amplification protocol (Q-TRAP) analysis was performed to measure telomerase activity^48^. Briefly, proteins were extracted from whole 48 hpf embryos, previously microinjected with RNA, aptamers or 20 µM BIBR1532 (telomerase inhibitor, Santa Cruz Biotechnology, #203843), using ice-cold TRAPeze 1X CHAPS Lysis Buffer (Sigma Aldrich). A real-time Q-TRAP was performed with 0.1 μg protein extracts. To make the standard curve, a 1:10 dilution series of positive telomerase sample (HeLa cells) was used. Control samples were obtained by treating the cell extracts with 1 μg RNase at 37 °C for 20 min. Data was collected and converted into Relative Telomerase Activity (RTA) units by calculating: RTA of sample=10^(Ct sample−γint)/slope^. The standard curve obtained was y = −3.2295x + 23.802.

### In vivo luciferase assay

pCMV-Renilla plasmid, containing Renilla luciferase (Promega) and pGL3basic vector constructs that contained the *csf3b* promoter region of zebrafish followed by firefly luciferase, were microinjected together with indicated aptamers or *terc* into the yolk sac of single-cell stage embryos. After 48 h, tail sections were obtained, pooled (20 tails per pool), homogenized with a pellet pestle and centrifuged for 3 min at 10,000 g to remove cellular debris. Then, extracts were assayed for firefly and Renilla luciferase activity using the Dual Luciferase Reporter Assay System (Promega) on a Luminometer Optocomp I (MGM Instruments). The results were normalized with the Renilla activity^49^.

### In vitro DNA binding assay

One hundred bp sense and antisense 3´ biotin-tagged-DNA probes of zebrafish *csf3b* promoter region encompassing *terc*-binding sites (Sigma-Aldrich) were designed to measure the capacity of *terc* or aptamers to bind to these sequences^9^. For annealing, 25 µM of each probe were incubated in annealing buffer (10 mM Tris-HCl pH 7.5, 200 mM NaCl, 0.2 mM EDTA) at 95 °C for 4 min, 10 min at 70 °C and they were slowly cooled down to RT for 20 min to allow annealing. Then, dsDNA probes were bound to 10 µl of Dynabeads MyOne Streptavidin C1 magnetic beads (Invitrogen) for 15 min at RT. Probe excess was removed by washing beads 2 times with annealing buffer for 5 min. Then, 50 ng of luciferase (Promega), *terc* and aptamers were added and incubated at RT for 30 min in rotation. The beads-dsDNA-RNA complexes were washed 3 times at RT for 10 min and RNA was eluted by incubation in water at 95 °C for 5 min. The eluted RNAs were reverse transcribed with SuperScript IV VILO Master Mix (Invitrogen) or miScript II RT kit (Qiagen) following the manufacturer’s instructions, for *terc* and luciferase or for aptamers, respectively. The samples were subjected to quantitative-PCR (qPCR) for luciferase, *terc* and aptamers detection, under the same conditions. To be able to compare the expression cycles of *terc*, aptamers and luciferase, normalization was carried out previously. Normalization consisted of a primers efficiency calibration curve starting from 1 ng/µl of RNA, and serial dilutions of 0.1 ng/µl, 0.01 ng/µl and 0.001 ng/µl of every RNA. Luciferase primers are more efficient than *terc* primers in 1.3 cycles, *CR7* aptamer in 3.8 cycles, *T800* aptamer in 19.2 cycles and *T1000* and *T1000*_*mut*_ aptamers in 18.7 cycles.

### Aptamer-based chromatin immunoprecipitation

A Chromatin Isolation by RNA Purification (ChIRP) protocol^9^ was performed with some modifications. Briefly, 5×106 HL60 cells were treated with 3´biotinylated-human aptamers (1 uM final concentration) in the culture medium for 48 h. Cells were washed in PBS, crosslinked with 1% gultaraldehyde for 10 min at room temperature, followed by incubation with 1/10 volume of 1,25 M glycine and lysed in ChIRP lysis buffer. Chromatin was then sonicated to a DNA fragment size of 100-500 bp in a Bioruptor Pico (Diagenode) and 100 ug of chromatin was incubated for 1 h with with Dynabeads™ MyOne™ Streptavidin C1 magnetic beads (Thermo Fisher Scientific) at 37 °C. Beads:biotin-aptamer:chromatin adducts were captured by magnets (Thermo Fisher Scientific) and washed five times with ChIRP washing buffer. Beads were resuspended in DNA elution buffer, and the DNA was eluted with a cocktail of RNase A (Sigma-Aldrich) and RNase H (Epicenter). Eluted chromatin was treated with proteinase K for 45 min at 65 °C. DNA was then extracted with equal volume of phenol:chloroform:isoamyl alcohol and precipitated with ethanol overnight at −80 °C. Eluted DNA was subjected to qPCR for detection of *terc*bs-containing *CSF2* promoter or 3'UTR *SPI1* gene chromatin fragments.

### RNA pulldown

RNA pulldown experiments were performed as described^50^, with some modifications^9^. Biotin-labeled RNAs were in vitro transcribed using Biotin 3´ End DNA Labeling Kit (Thermo Scientific), to incorporate 1-3 biotinylated ribonucleotides onto 3´ end of DNA/RNA strands, following the manufacturer’s instructions. Three micrograms of biotinylated RNA were heated to 70 °C for 5 min and put on ice for 2 min. An equal volume of 2x RNA structure buffer (20 mM Tris pH 7, 0.2 M KCl, 20 mM MgCl_2_) was added and then shifted to RT to allow RNA secondary structure formation. Folded RNAs were incubated for 1 h at 4 °C with rotation with 60 µl of washed Dynabeads MyOne Streptavidin C1 magnetic beads (Invitrogen). Protein extract from 5 dpf larvae was obtained by homogenization in RIP buffer (25 mM Tris pH 7.4, 150 mM KCl, 0.5 mM DTT, 0.5% NP-40), centrifuged for 20 min at 4 °C and the supernatant was pre-cleared 1 h with 30 µl of beads at 4 °C under rotation. Then, three miligrams of pre-cleared protein were incubated with biotinylated RNAs-beads complexes for 4 h at 4 °C with rotation. Complexes were magnet-captured, washed with RIP buffer five times at 4 °C for 5 min and boiled in 2x Laemmli buffer (Sigma) at 90 °C for 10 min for protein elution. Eluted proteins were subjected to polyacrylamide gel electrophoresis, wet transferred to a nylon membrane (GE Healthcare) and analyzed by Western blot. Membranes were incubated for 1 h with TTBS (Tween-Tris-buffered saline) containing 5% (w/v) skimmed dried milk powder and immunoblotted using anti-phospho-Serine 5 RNA polymerase II CTD repeat YSPTSPS mouse antibodies (dilution 1:1000) 16 h at 4 °C (pS5 RNA pol II, ab5408, Abcam). Blots were then washed with TTBS and incubated for 1 h at room temperature with the secondary HRP-conjugated antibody (dilution 1:1000) in 5% (w/v) skimmed milk in TTBS. After repeated washes, the signal was detected with the enhanced chemiluminescence reagent and ChemiDoc XRS (Biorad).

### Zebrafish model of poikiloderma with neutropenia

To generate a zebrafish model of poikiloderma with neutropenia, the *usb1* (U6 SnRNA Biogenesis Phosphodiesterase 1) gene^31,32^ was inhibited by CRISPR-Cas9 technology. CRISPR RNA (crRNA) was obtained from IDT with the following target sequence was used: *usb1* 5′-GGAAGCTCTTCATCACCTTCAGG-3′. It was resuspended in duplex buffer at 100 μM and 1 μl was incubated with 1 μl of trans-activating CRISPR RNA (tracrRNA, 100 μΜ) at 95 °C for 5 min and 5 min at RT to form the complex. One μl of these complexes was mixed with 0.25 μl of recombinant Cas9 (10 mg/ml) and 3.75 μl of duplex buffer. The crRNA mix was then microinjected into the yolk sac of one-cell stage zebrafish embryos using a microinjector (Narishige) (1 nl per embryo). Neutrophils were counted at 3 dpf.

### iPSC culture and differentiation towards hematopoietic lineage and CFU assay

iPSC were obtained by Dr Sunnet Agarwal (Department of Medical Oncology, Dana-Farber Cancer Institute, Boston, MA.) who has custody of confirmation of IRB approval and patient consents.

iPSC lines were maintained undifferentiated in a 6 cm plates treated with Matrigel (Corning) and mTeSR™ Plus medium (Stem Cell Technologies). The medium was changed daily or every two days, and cells were split weekly by dissociation with 200 U/ml of collagenase IV (Invitrogen). iPSC cultures were visualized daily by phase-contrast microscopy.

For hematopoietic differentiation, undifferentiated iPSC at 70-80% confluence were treated with Matrigel (Corning) 24 h before starting differentiation. To generate EBs, the iPSC were treated with collagenase IV and scraped off. They were then transferred to 6-well low-attachment plates (Corning) to allow EB formation by incubation in differentiation medium consisting of KnockOut™ Dulbecco’s modified Eagle’s medium (ThermoFisher) supplemented with 20% non-heat-inactivated fetal bovine serum, 1% nonessential amino acids, 1 mM glutamine and 0.1 mM β-mercaptoethanol. The medium was changed the following day (day 1) with the same differentiation medium supplemented with hematopoietic cytokines: 300 ng/ml stem cell factor (R&D), 300 ng/ml FMS-like tyrosine kinase-3 ligand (R&D), 10 ng/ml interleukin-3 (IL-3, R&D), 10 ng/ml IL-6 (R&D), 50 ng/ml GM-CSF (R&D) and 25 ng/ml bone morphogenetic protein-4 (Miltenyi)^51–53^. At day 7, aptamers are added to the medium and refreshed every 2 days. At day 15 of development, EBs were dissociated, and single-cell suspensions were stained with anti-CD34-fluorescein isothiocyanate, anti-CD31-phycoerythrin and anti-CD45-allophycocyanin antibodies (all from Becton Dickinson) and analyzed by flow cytometry.

CFU assays were performed by plating 100,000 cells from EBs at day 15 into methylcellulose H4434 culture medium (Stem Cell Technologies). The cells were incubated at 37 °C in a 5% CO_2_ humidified atmosphere and colonies were counted at day 14 of the CFU assay using standard morphological criteria^54–56^. The iPSC workflow is depicted in Fig. 5b.

### Modeling of three-dimensional structures of aptamers and *TERC*

Prediction and modelling of tertiary RNA structure using the RNAComposer system (http://rnacomposer.cs.put.poznan.pl/)^57–60^. The structure was processed with Jmol application, an open-source Java viewer for chemical structures in 3D.

### Statistical analysis

Statistical analysis was performed by using GraphPad Prism 8. Data were analyzed by analysis of variance (ANOVA) or mixed-effect analysis (see Figure legends for further details).

### Reporting summary

Further information on research design is available in the Nature Portfolio Reporting Summary linked to this article.

### Supplementary Information

Supplementary Information

Reporting Summary

### Source data

Source Data

## Data Availability

Source data are provided with this paper. All data supporting the findings described in this manuscript are available in the article and in the Supplementary Information and from the corresponding author upon request. Source data are provided with this paper.
