# Supplementary material for: Telomerase RNA-based aptamers restore defective myelopoiesis in congenital neutropenic syndromes
Source: Nat Commun. 2023 Sep 22;14:5912. doi: 10.1038/s41467-023-41472-7 (PMC10516865; doi:10.1038/s41467-023-41472-7)
Supplement: Supplementary file 1 — Supplementary Information [file 41467_2023_41472_MOESM1_ESM.pdf]

a

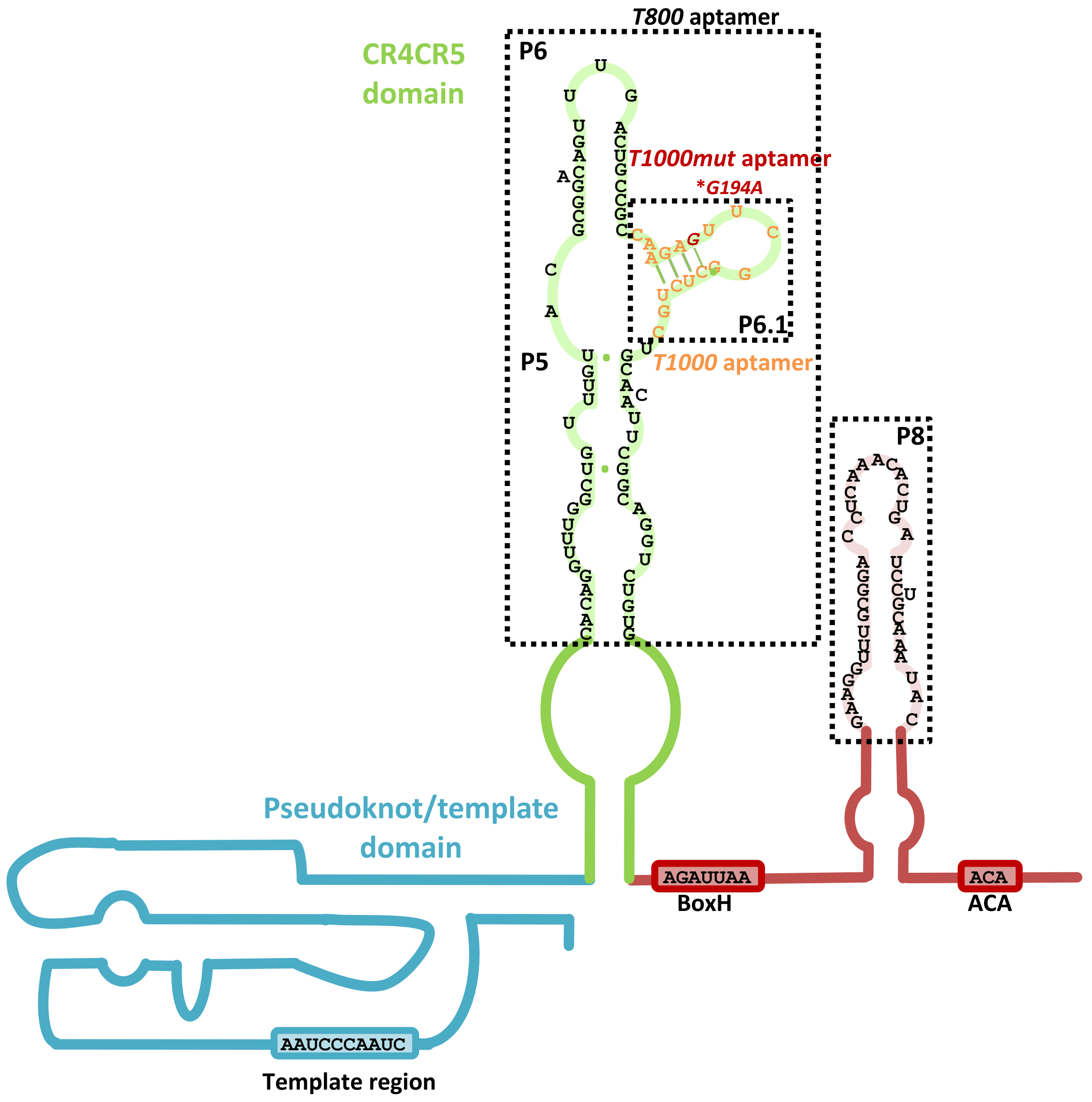

b

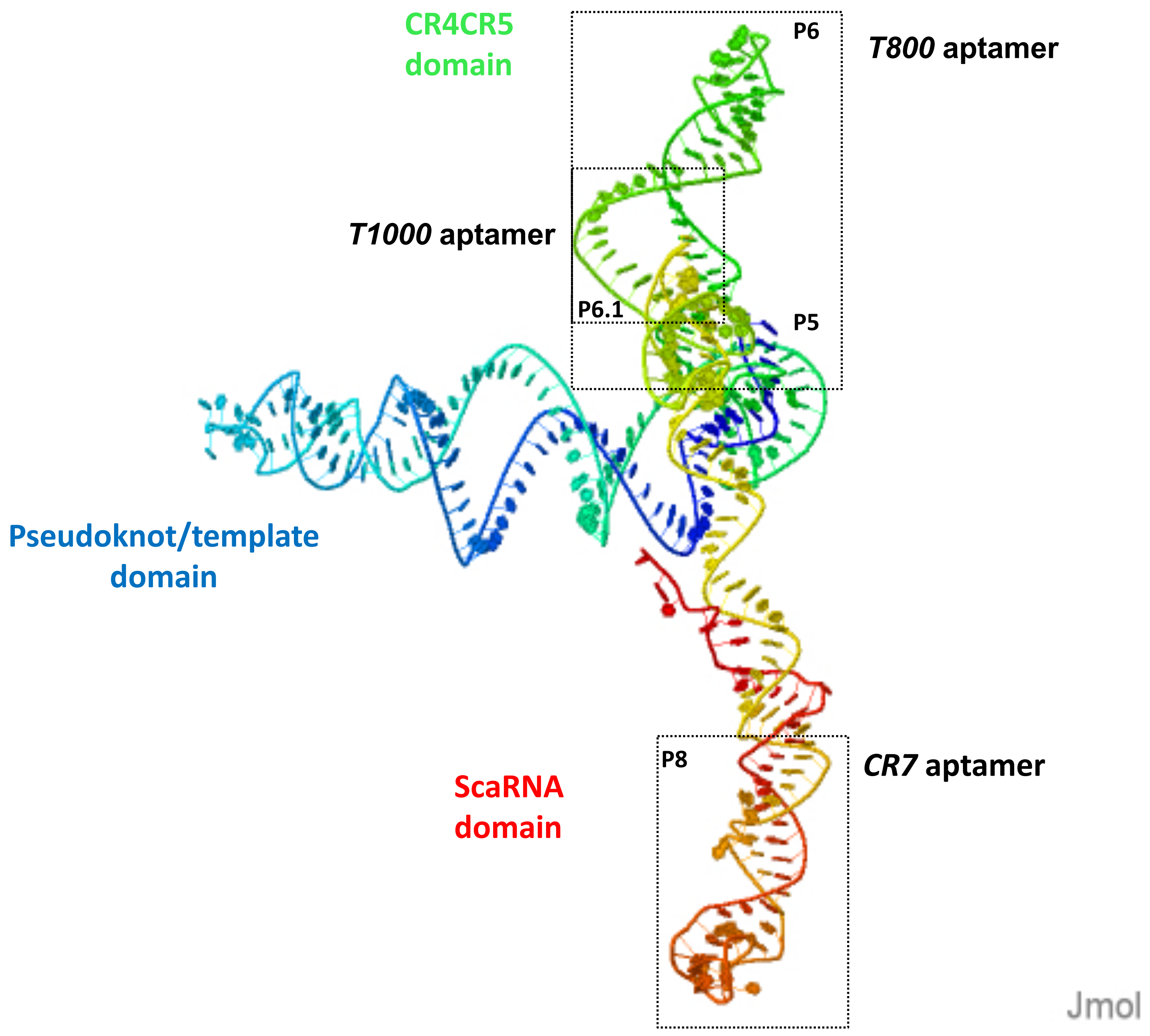

c

Aptamers	Zebrafish	Human	Mouse
T800	CACAGGUUUGGCUGUUUGUACGCGGACAGUUUGA CUGCCGCCAAGAGUUCGGCUCUGCUGCACAUUCGG CAGGUCUGUG (79 nt)	CCCGCCUGGAGGCCGCGGUCGGCCCGGGGCU UCUCCGGAGGCACCCACUGCCACCGCGAAGAG UUGGGCUCUGUCAGCCGCGGG (84 nt)	-
CR7	GAAGGUUUGCGGACCUCAAACACUGAUCCUGCAAA UAC (38 nt)	CCCCGCGCGCGGCGCGAUUCCCUGAGCUGUG GGACGUGCACCCAGG (46 nt)	-
T1000	CAAGAGUUCGGCUCUGC (17 nt)	AAGAGUUGGGCUCUG (15 nt)	GAAGAGCUCGCCUCUG (16 nt)
T1000mut	CAAGAAUUCGGCUCUGC (17 nt)	AAGAAUUGGGCUCUG (15 nt)	GAAGAACUCGCCUCUG (16 nt)

d

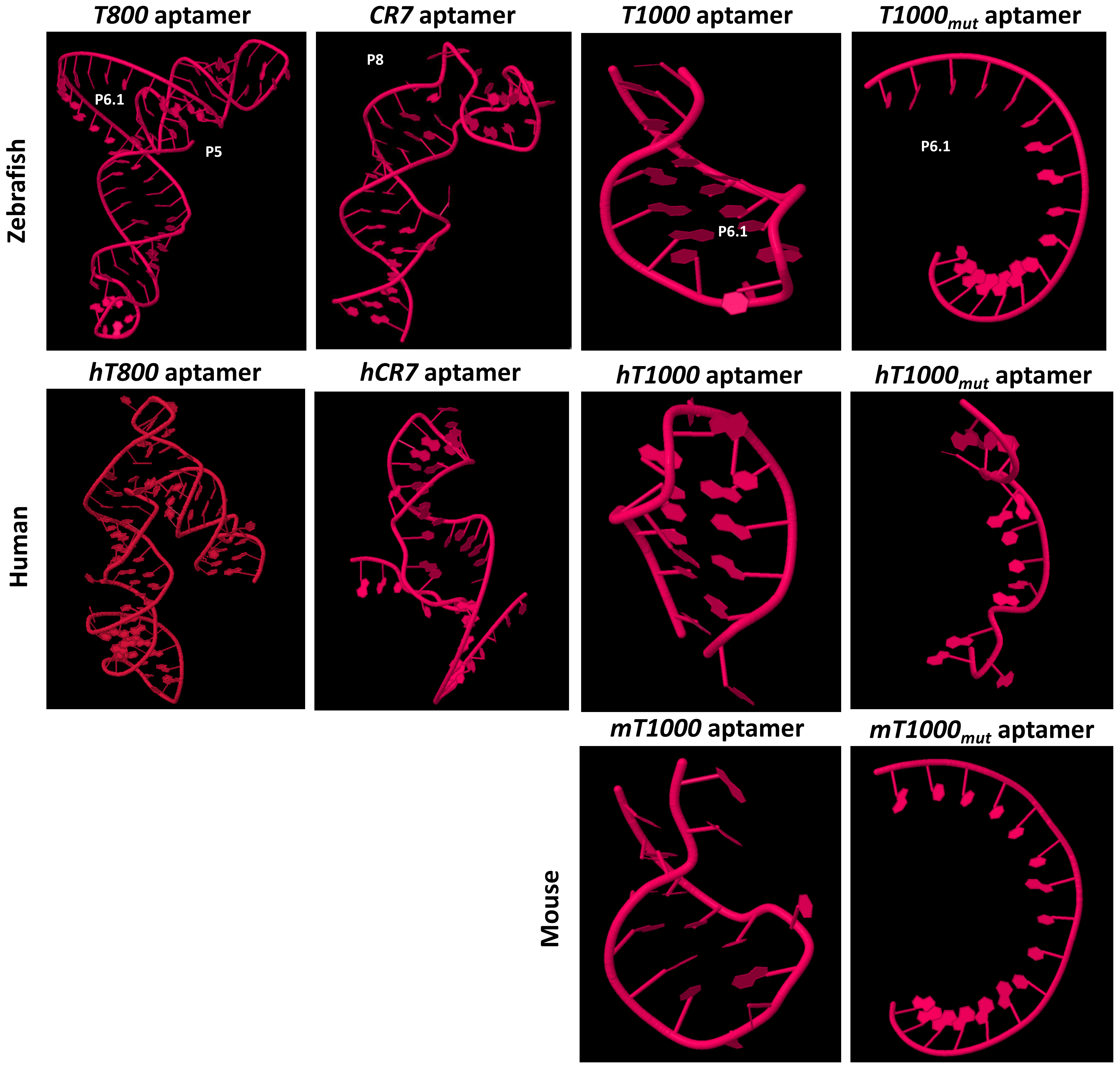

Figure S1. Aptamer design. (a) Zebrafish *terc* secondary structure highlighting aptamers position inside the CR4/CR5 domain and showing the mutation involved in aplastic anemia (G194A). (b) Zebrafish *terc* tertiary RNA structure highlighting aptamer localization. (c) Aptamer sequence in zebrafish, mouse and human and their length showing in red font the AA mutation. (d) Zebrafish, human and mouse aptamers 3D structure**.** Prediction of tertiary structure of aptamers and *TERC* was obtained with the RNAComposer system.

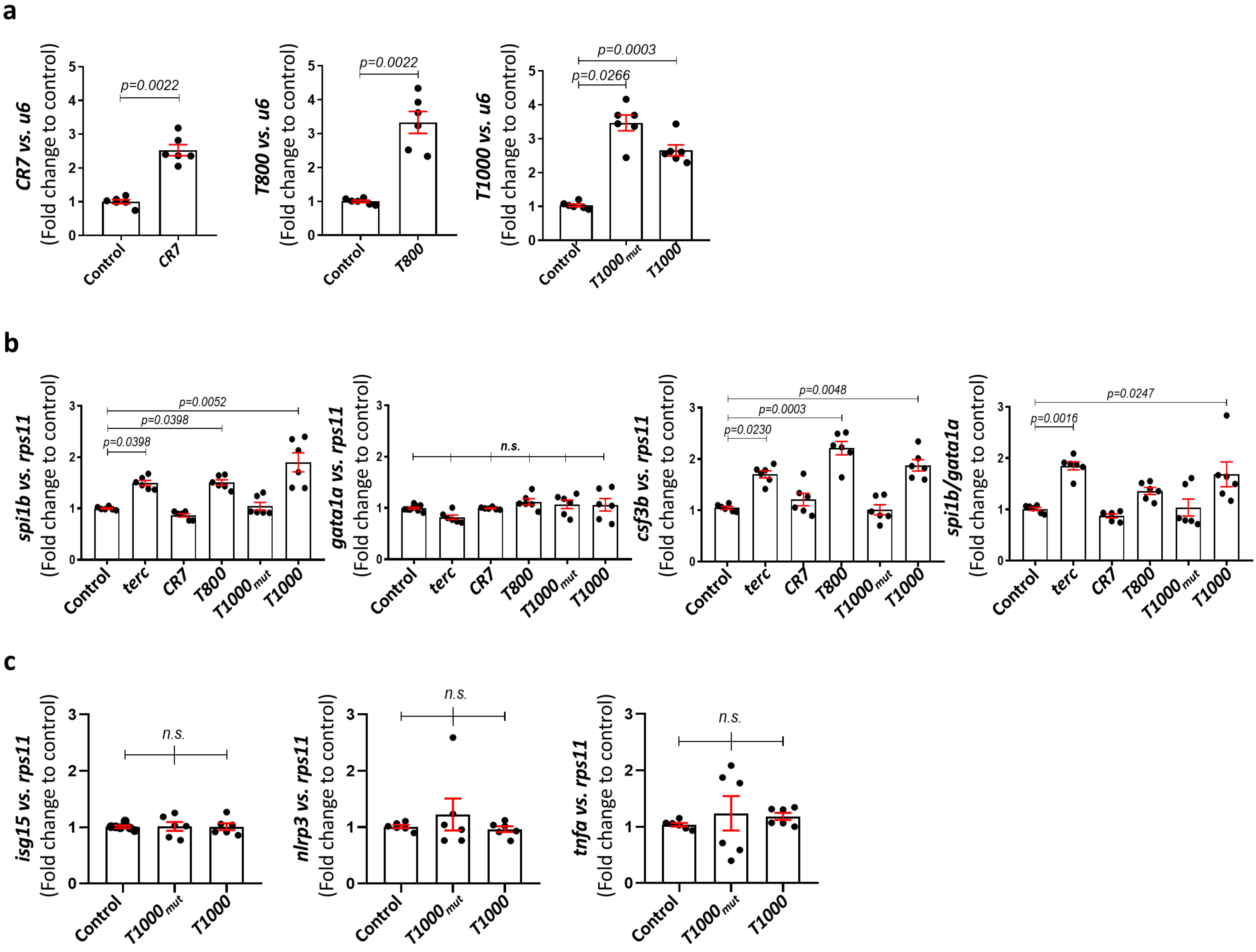

Figure S2. Aptamers do not induce the expression of pro-inflammatory genes and do not affect neutrophil function. (a) Levels of *CR7*, *T800* and *T1000* aptamers measured by RT-qPCR. Gene expression was normalized to *u6* gene and relative to control. Data are average of 6 biologically independent samples represented by dots. Bars represents mean ± SEM. For *CR7* and *T800*, statistical analysis according to two-tailed Mann-Whitney test (95% confidence interval); and for *T1000*, statistical analysis according to Kruskal-Wallis followed by uncorrected Dunn's test (95% confidence interval).(b) mRNA levels determined by RT-qPCR of the myeloid genes *spi1b*, *gata1a* and *csf3b* and *spi1b*/*gata1a* ratio in 48 hpf larval tails microinjected with the indicated aptamers. The expression was normalized to *rps11* and relative to control sample. Data are average of 6 biologically independent samples represented by dots. Bars represents mean ± SEM. *n.s.*, not significant, p > 0.05 according to Kruskal-Wallis followed by uncorrected Dunn's test (95% confidence interval).(c) The levels of *isg15*, *nlrp3* and *tnfa* were measured by RT-qPCR. Gene expression was normalized to *rps11* gene and relative to control. Data are average of 6 biologically independent samples represented by dots. Bars represents mean ± SEM. *n.s.*, not significant, p > 0.05 according to Kruskal-Wallis followed by uncorrected Dunn's test (95% confidence interval).

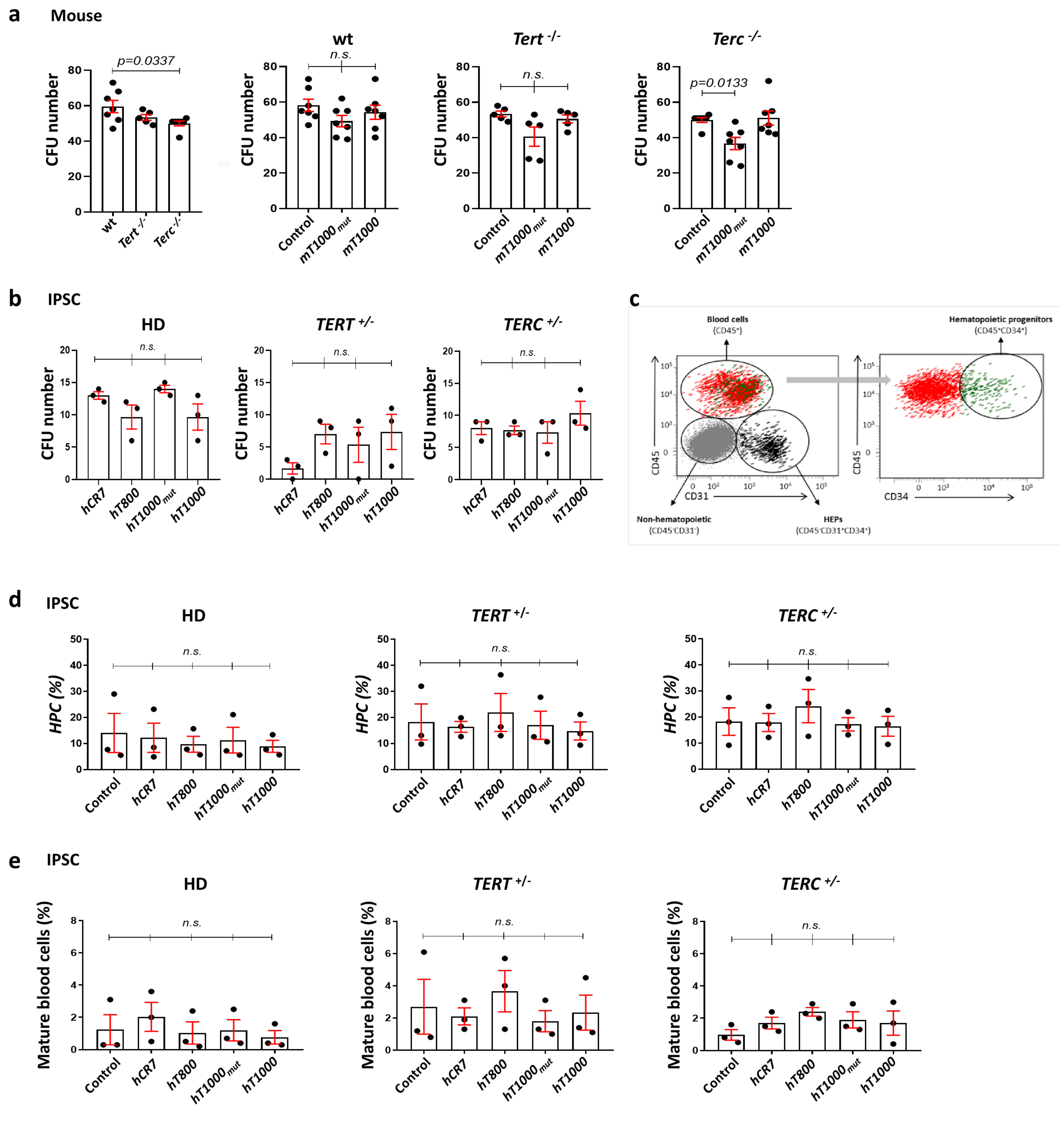

Figure S3. Hematopoietic differentiation of mouse bone marrow cells and EBs-derived iPSC treated with aptamers. (a) CFU quantification of wildtype, *Tert*$^{-/-}$ and *Terc*$^{-/-}$ mouse genotypes after aptamer treatment. Data are average of at least 5 biologically independent samples represented by dots (wt, n=7; *Tert*-/-, n=5; *Terc*-/-, n=7). Bars represent mean ± SEM. *n.s.*, not significant, p > 0.05, according to Kruskal-Wallis followed by Dunn's multiple comparison test (95% confidence interval) .(b) CFU quantification of HD, TERT-deficient and *TERC*-deficient EBs at day 14 after hematopoietic differentiation. Data are average of 3 biologically independent samples represented by dots. Bars represent mean ± SEM. *n.s.*, not significant, p > 0.05, according to Kruskal-Wallis followed by Dunn's multiple comparison test (95% confidence interval).(c) Dot plot and identification of different cell populations in EBs by flow cytometry. CD31+ HEP (bipotential precursors of hematopoietic and endothelial cells), CD45+ CD34+ HPC, and CD45+ mature blood cells.(d)Quantification of hematopoietic progenitor cells (HPC) population (CD31^{-} /CD34^{+} /CD45^{+}) in EBs of 15 days treated with the indicated aptamers. Data are average of 3 biologically independent samples represented by dots. Bars represent mean ± SEM for each group. *n.s.*, not significant, p > 0.05, according to Kruskal-Wallis followed by Dunn's multiple comparison test (95% confidence interval). (e) Quantification of mature blood cells population (CD34^{-} /CD45^{+}) in EBs of 15 days treated with the indicated aptamers. Data are average of 3 biologically independent samples represented by dots. Bars represent mean ± SEM for each group. *n.s.*, not significant, p > 0.05, according to Kruskal-Wallis followed by Dunn's multiple comparison test (95% confidence interval).

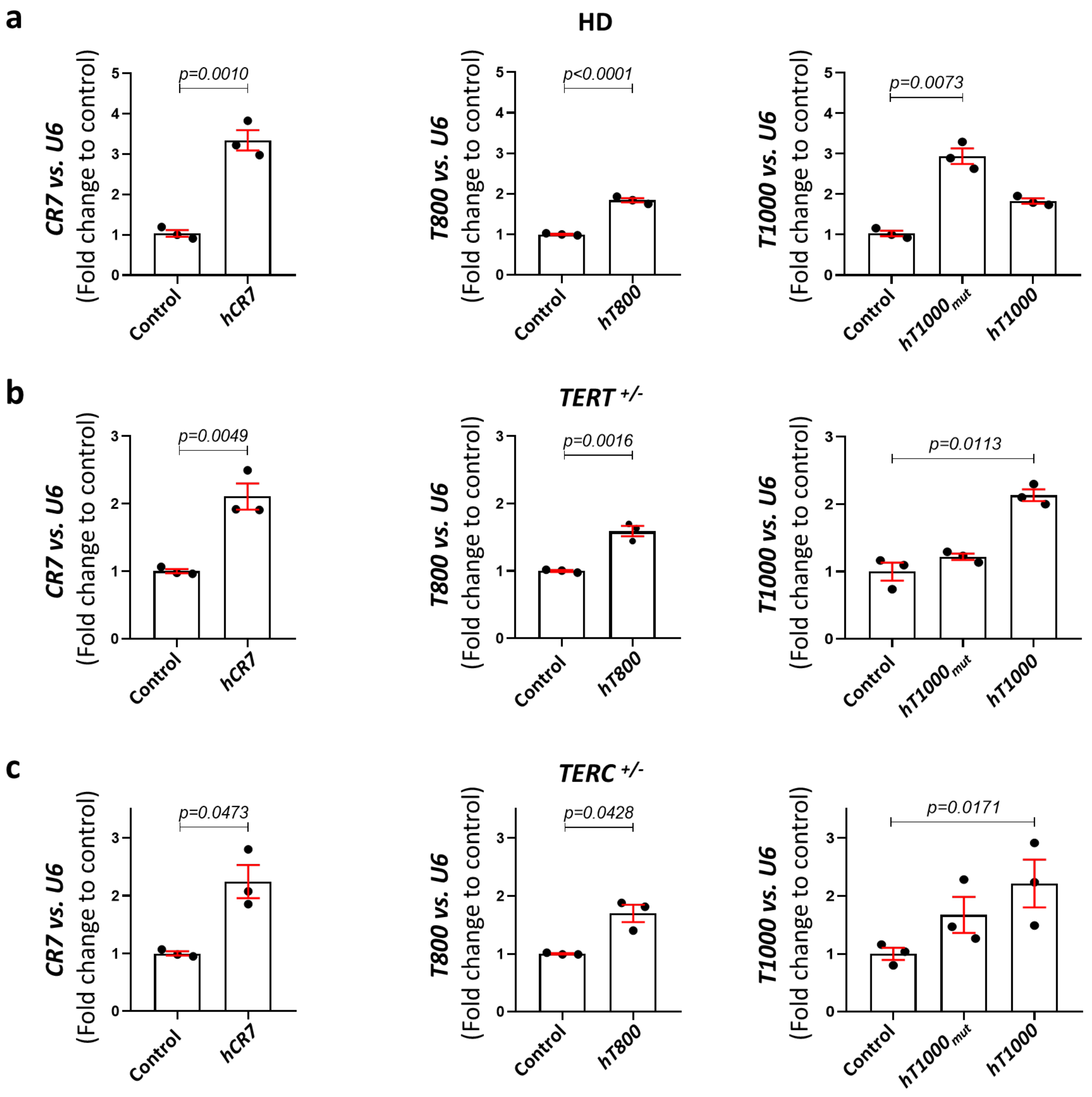

Figure S4. Aptamer quantification in human iPSC. The levels of *CR7*, *T800* and *T1000* aptamers was measured by RT-qPCR in HD, TERT-deficient and *TERC*-deficient iPSC. Gene expression was normalized to U6 gene and relative to control. (a). Data are average of 3 biologically independent samples represented by dots. Bars represent mean ± SEM for each group. For *CR7* and *T800*, statistical analysis according to two-tailed unpaired t-test (95% confidence interval); and for *T1000*, statistical analysis according to Kruskal-Wallis followed by uncorrected Dunn's test (95% confidence interval). (b).Data are average of 3 biologically independent samples represented by dots. Bars represent mean ± SEM for each group. For *CR7* and *T800*, statistical analysis according to two-tailed unpaired t-test (95% confidence interval); and for *T1000*, statistical analysis according to Kruskal-Wallis followed by uncorrected Dunn's test (95% confidence interval). (c). Data are average of 3 biologically independent samples represented by dots. Bars represent mean ± SEM for each group. For *CR7* and *T800*, statistical analysis according to two-tailed unpaired t-test with Welch's correction (95% confidence interval); and for *T1000*, statistical analysis according to Kruskal-Wallis followed by uncorrected Dunn's test (95% confidence interval).

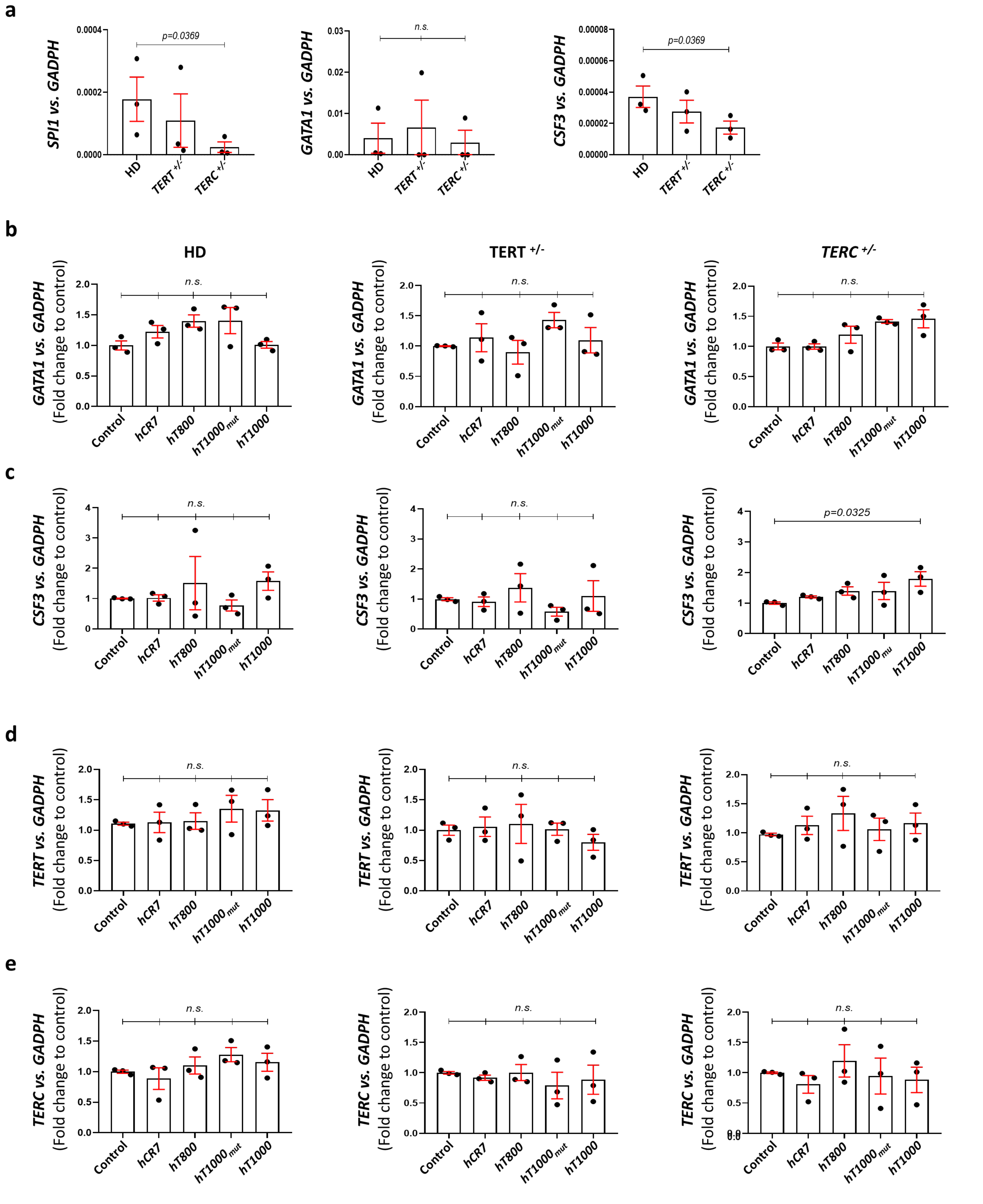

Figure S5. Gene expression profile of embryonic bodies treated with aptamers. The mRNA levels of *SPI1 (a), GATA1* (a, b), *CSF3* (a, c), *TERT (d)* and *TERC* (e) were determined by RT-qPCR in HD, TERT-deficient and *TERC*-deficient EBs treated with the indicated aptamers. Gene expression was normalized to *GAPDH* (a-e) and relative to control sample (b-e). (a). Data are average of 3 biologically independent samples represented by dots. Bars represent mean ± SEM for each group. *n.s.*, not significant, $p > 0.05$, according to Kruskal-Wallis followed by uncorrected Dunn's test (95% confidence interval). (b). Data are average of 3 biologically independent samples represented by dots. Bars represent mean ± SEM for each group. *n.s.*, not significant, $p > 0.05$ according to Kruskal-Wallis followed by corrected Dunn's test (95% confidence interval). (c): Data are average of 3 biologically independent samples represented by dots. Bars represent mean ± SEM for each group. *n.s.*, not significant, $p > 0.05$, according to Kruskal-Wallis followed by corrected Dunn's test (95% confidence interval).(d). Data are average of 3 biologically independent samples represented by dots. Bars represent mean ± SEM for each group. *n.s.*, not significant, $p > 0.05$, according to Kruskal-Wallis followed by corrected Dunn's test (95% confidence interval). (e) Data are average of 3 biologically independent samples represented by dots. Bars represent mean ± SEM for each group. *n.s.*, not significant, $p > 0.05$, according to Kruskal-Wallis followed by corrected Dunn's test (95% confidence interval).

Stage \ Condition	WT (n=14)	*+T800* (n=15)	*+T1000mut* (n=14)	+T1000 (n=15)
**22 hpf / 26 somites**<br>- otholites	*present*	*present*	*present*	*present*
**24-25 hpf / Prim-5, -6**<br>- spontaneous side-to-side contractions<br>- onset of retinal and skin pigmentation<br>- start of heartbeat<br>- presence of blood cells in the yolk	*present*<br>*present*<br>*present*<br>*present*	*present*<br>*present*<br>*present*<br>*present*	*present*<br>*present*<br>*present*<br>*present*	*present*<br>*present*<br>*present*<br>*present*
**30-31 hpf / Prim-15, -16**<br>- pigmented retina<br>- caudal artery 50% tail<br>- braided caudal vein<br>- onset of blood circulation<br>- straight tail<br>- head-trunk axis (HTA)= 95º<br>- shallow bud of pectoral fin	*present*<br>*present*<br>*present*<br>*present*<br>*present*<br>*present*<br>*present*	*present*<br>*present*<br>*present*<br>*present*<br>*present*<br>*present*<br>*present*	*present*<br>*present*<br>*present*<br>*present*<br>*present*<br>*present*<br>*present*	*present*<br>*present*<br>*present*<br>*present*<br>*present*<br>*present*<br>*present*

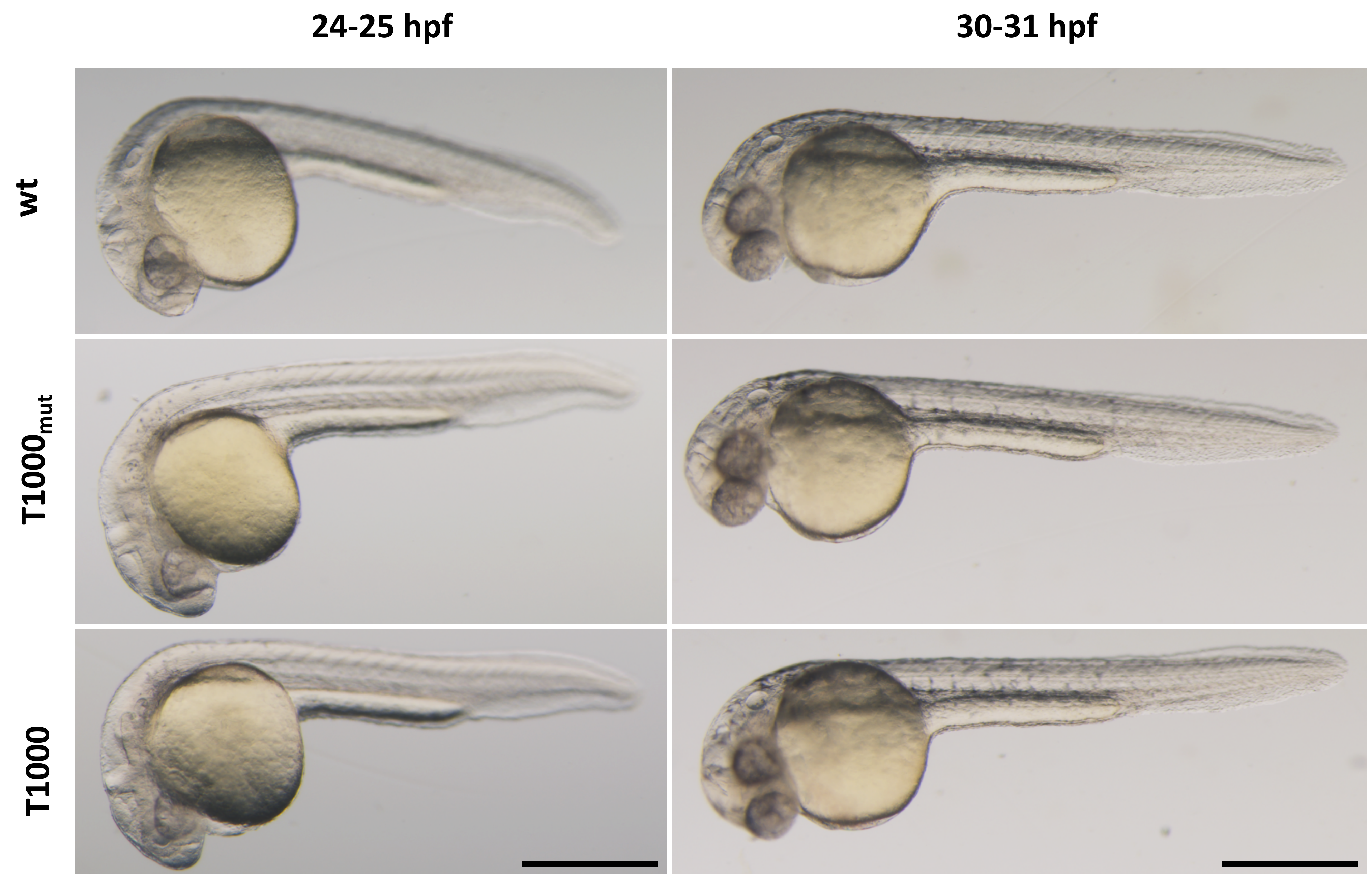

Table I. (a) Assessment of the toxicity of aptamers by analysing their effect on development. Based on Kimmel CB, Ballard WW, Kimmel SR, Ullmann B, Schilling TF. Stages of zebrafish embryonic development. Dev Dyn. 1995 Jul;203(3):253-310. Doi: 10.1002/aja.1002030302. PMID: 8589427. (b) Representative image of zebrafish larvae development with indicated treatment of aptamer and developmental time. BAR SCALE:500 um
