# Supplementary material for: Telomerase RNA-based aptamers restore defective myelopoiesis in congenital neutropenic syndromes
Source: Nat Commun. 2023 Sep 22;14:5912. doi: 10.1038/s41467-023-41472-7 (PMC10516865; doi:10.1038/s41467-023-41472-7)
Supplement: Supplementary file 2 — Reporting Summary [file 41467_2023_41472_MOESM2_ESM.pdf]

nature portfolio

Corresponding author(s): María L. Cayuela

Last updated by author(s): Jul 3, 2023

Reporting Summary

Nature Portfolio wishes to improve the reproducibility of the work that we publish. This form provides structure for consistency and transparency in reporting. For further information on Nature Portfolio policies, see our Editorial Policies and the Editorial Policy Checklist.

Statistics

For all statistical analyses, confirm that the following items are present in the figure legend, table legend, main text, or Methods section.

n/a	Confirmed	
☐	☒	The exact sample size (*n*) for each experimental group/condition, given as a discrete number and unit of measurement
☐	☒	A statement on whether measurements were taken from distinct samples or whether the same sample was measured repeatedly
☐	☒	The statistical test(s) used AND whether they are one- or two-sided<br>*Only common tests should be described solely by name; describe more complex techniques in the Methods section.*
☒	☐	A description of all covariates tested
☐	☒	A description of any assumptions or corrections, such as tests of normality and adjustment for multiple comparisons
☐	☒	A full description of the statistical parameters including central tendency (e.g. means) or other basic estimates (e.g. regression coefficient) AND variation (e.g. standard deviation) or associated estimates of uncertainty (e.g. confidence intervals)
☐	☒	For null hypothesis testing, the test statistic (e.g. *F*, *t*, *r*) with confidence intervals, effect sizes, degrees of freedom and *P* value noted<br>*Give P values as exact values whenever suitable.*
☒	☐	For Bayesian analysis, information on the choice of priors and Markov chain Monte Carlo settings
☒	☐	For hierarchical and complex designs, identification of the appropriate level for tests and full reporting of outcomes
☒	☐	Estimates of effect sizes (e.g. Cohen's *d*, Pearson's *r*), indicating how they were calculated

Our web collection on statistics for biologists contains articles on many of the points above.

Software and code

Policy information about availability of computer code

Data collection	not applicable
Data analysis	Three-dimensional folds of RNA structures : https://rnacomposer.cs.put.poznan.pl/<br>Sytadistical analysis:GraphPad Prism software

For manuscripts utilizing custom algorithms or software that are central to the research but not yet described in published literature, software must be made available to editors and reviewers. We strongly encourage code deposition in a community repository (e.g. GitHub). See the Nature Portfolio guidelines for submitting code & software for further information.

Data

Policy information about availability of data

All manuscripts must include a data availability statement. This statement should provide the following information, where applicable:

- Accession codes, unique identifiers, or web links for publicly available datasets
- A description of any restrictions on data availability
- For clinical datasets or third party data, please ensure that the statement adheres to our policy

not applicable

Human research participants

Policy information about studies involving human research participants and Sex and Gender in Research.

Reporting on sex and gender	not applicable
Population characteristics	not applicable
Recruitment	not applicable
Ethics oversight	not applicable

Note that full information on the approval of the study protocol must also be provided in the manuscript.

Field-specific reporting

Please select the one below that is the best fit for your research. If you are not sure, read the appropriate sections before making your selection.

- [x] Life sciences
- [ ] Behavioural & social sciences
- [ ] Ecological, evolutionary & environmental sciences

For a reference copy of the document with all sections, see nature.com/documents/nr-reporting-summary-flat.pdf

Life sciences study design

All studies must disclose on these points even when the disclosure is negative.

Sample size	Samples sizes were based on the experience of the autors with molecular and in vivo studie as publised in many studies. For animal models, exeriments were designed to detect differences between treatment groups or genotype-dependent effects at 80% power (p=0.05)
Data exclusions	The levels of every aptamer were checked in zebrafish larvae by RT-qPCR in every single experiment and if they were not detected, the experiment was discarded.
Replication	the experiment were perfomed al least three times
Randomization	Zebrafish larvae were tandomly assigned to each group by simple randomization
Blinding	Blinding was not possible in the majority of the lab experiments becouse they were done by one single person

Reporting for specific materials, systems and methods

We require information from authors about some types of materials, experimental systems and methods used in many studies. Here, indicate whether each material, system or method listed is relevant to your study. If you are not sure if a list item applies to your research, read the appropriate section before selecting a response.

Materials & experimental systems

n/a	Involved in the study	
	x	Antibodies
	x	Eukaryotic cell lines
x		Palaeontology and archaeology
	x	Animals and other organisms
x		Clinical data
x		Dual use research of concern

Methods

n/a	Involved in the study	
x		ChIP-seq
	x	Flow cytometry
x		MRI-based neuroimaging

Antibodies

Antibodies used	anti-phospho-Serine 5 RNA polymerase II CTD repeat YSPTSPS mouse antibodies (dilution 1:1000) 16 h at 4°C (pS5 RNA pol II, ab5408, Abcam)<br>anti-CD34-fluorescein isothiocyanate, anti-CD31-phycoerythrin and anti-CD45-allophycocyanin antibodies (all from Becton Dickinson)
Validation	Comercially available antibody was validated by manufaturers and in previous paper PMID: 34353901

Eukaryotic cell lines

Policy information about cell lines and Sex and Gender in Research

Cell line source(s)	IPSC were provided by Dr Argarwal.Boston Children's Hospital. Dana Farber Cancer Institute<br>HL60 cell line was provided by cell culture facility of Universidad de Murcia
Authentication	None of the IPSC lines were identified.
Mycoplasma contamination	HL60 was negative for mycoplasma contamination<br>IPSC were not checked for mycoplasma contamination
Commonly misidentified lines<br>(See ICLAC register)	NO

Animals and other research organisms

Policy information about studies involving animals; ARRIVE guidelines recommended for reporting animal research, and Sex and Gender in Research

Laboratory animals	zebrafish larvae
Wild animals	not applicable
Reporting on sex	not applicable
Field-collected samples	not applicable
Ethics oversight	The performed experiments comply with the Guidelines of the European Union Council (Directive 2010/63/EU) and the Spanish RD 53/2013. Experiments and procedures were performed as approved by the Bioethical Committees of the University of Murcia (approval numbers #75/2014, #216/2014 and 395/2017) and Bioethical Committees of the Hospital Universitario Virgen de la Arrixaca (A13211202)

Note that full information on the approval of the study protocol must also be provided in the manuscript.

Flow Cytometry

Plots

Confirm that:

- [x] The axis labels state the marker and fluorochrome used (e.g. CD4-FITC).
- [x] The axis scales are clearly visible. Include numbers along axes only for bottom left plot of group (a 'group' is an analysis of identical markers).
- [x] All plots are contour plots with outliers or pseudocolor plots.
- [x] A numerical value for number of cells or percentage (with statistics) is provided.

Methodology

Sample preparation	For hematopoietic differentiation, undifferentiated iPSC at 70-80% confluence were treated with Matrigel (Corning) 24 hours before starting differentiation. To generate EBs, the iPSC were treated with collagenase IV and scraped off. They were then transferred to 6-well low-attachment plates (Corning) to allow EB formation by incubation in differentiation medium consisting of KnockOut™ Dulbecco's modified Eagle's medium (ThermoFisher) supplemented with 20% non-heat-inactivated fetal bovine serum, 1% nonessential amino acids, 1 mM glutamine and 0.1 mM β-mercaptoethanol. The medium was changed the following day (day 1) with the same differentiation medium supplemented with hematopoietic cytokines: 300 ng/ml stem cell factor (R&D), 300 ng/ml FMS-like tyrosine kinase-3 ligand (R&D), 10 ng/ml interleukin-3 (IL-3, R&D), 10 ng/ml IL-6 (R&D), 50 ng/ml GM-CSF (R&D) and 25 ng/ml bone morphogenetic protein-4 (Miltenyi) 51-53. At day 7, aptamers are added to the medium and refreshed every 2 days. At day 15 of development, EBs were dissociated, and single-cell suspensions were stained with anti-CD34-fluorescein isothiocyanate, anti-CD31-phycoerythrin and anti-CD45-allophycocyanin antibodies (all from Becton Dickinson) and analyzed by flow cytometry.
Instrument	LsRFortessa x20
Software	BD FACSDivaTM. v8.0
Cell population abundance	not applicable
Gating strategy	All the gating strategy has been previously described in DOI: 10.1073/pnas.2015528118 .

- [x] Tick this box to confirm that a figure exemplifying the gating strategy is provided in the Supplementary Information.
